# Molecular Research on Oral Diseases and Related Biomaterials: A Journey from Oral Cell Models to Advanced Regenerative Perspectives

**DOI:** 10.3390/ijms23095288

**Published:** 2022-05-09

**Authors:** Thorsten Steinberg, Martin Philipp Dieterle, Pascal Tomakidi

**Affiliations:** Division of Oral Biotechnology, Center for Dental Medicine, Medical Center University of Freiburg, Faculty of Medicine, University of Freiburg, Hugstetter Str. 55, 79106 Freiburg, Germany; martin.dieterle@uniklinik-freiburg.de (M.P.D.); pascal.tomakidi@uniklinik-freiburg.de (P.T.)

**Keywords:** carcinogenesis, cell transformation, tissue homeostasis, mechanotransduction, non-woven, mesenchymal stem cells

## Abstract

Oral diseases such as gingivitis, periodontitis, and oral cancer affect millions of people worldwide. Much research has been conducted to understand the pathogenetic mechanisms of these diseases and translate this knowledge into therapeutics. This review aims to take the reader on a journey from the initial molecular discoveries to complex regenerative issues in oral medicine. For this, a semi-systematic literature search was carried out in Medline and Web of Science databases to retrieve the primary literature describing oral cell models and biomaterial applications in oral regenerative medicine. First, an in vitro cell model of gingival keratinocytes is discussed, which illustrates patho- and physiologic principles in the context of oral epithelial homeostasis and carcinogenesis and represents a cellular tool to understand biomaterial-based approaches for periodontal tissue regeneration. Consequently, a layered gradient nonwoven (LGN) is described, which demonstrates that the key features of biomaterials serve as candidates for oral tissue regeneration. LGN supports proper tissue formation and obeys the important principles for molecular mechanotransduction. Furthermore, current biomaterial-based tissue regeneration trends, including polymer modifications, cell-based treatments, antimicrobial peptides and optogenetics, are introduced to represent the full spectrum of current approaches to oral disease mitigation and prevention. Altogether, this review is a foray through established and new concepts in oral regenerative medicine and illustrates the process of knowledge translation from basic molecular and cell biological research to future clinical applications.

## 1. Introduction

Diseases of the oral cavity affect millions of people worldwide and lead to considerable morbidity and mortality [1,2]. From a clinicopathological perspective, these diseases can be classified as infectious/inflammatory (e.g., stomatitis, pulpitis, periodontitis), autoimmune/dysimmune (e.g., oral manifestations of Systemic Lupus Erythematosus or Sjögren syndrome), neoplastic (e.g., squamous dysplasia of the oral cavity or oral squamous cell carcinoma (OSCC)), traumatic, or congenital (e.g., odontogenic cysts) [3,4,5,6,7,8]. In 2020, 377,713 new cases of cancer of the lip and oral cavity and a total of 177,757 deaths of both sexes and all ages due to this disease were registered by the International Agency for Research on Cancer (IARC), World Health Organization (WHO) (https://gco.iarc.fr/today/fact-sheets-cancers (accessed on 19 January 2022)). According to the Global Burden of Disease Study, severe periodontal disease was the 11th most prevalent health condition in the world. Herein, the prevalence of periodontal disease, comprising gingivitis and periodontitis, was reported to range from 20% to 50% around the world (The Global Burden of Diseases, Injuries, and Risk Factors Study 2017, GBD 2017) [9]. These numbers illustrate the urgent need for basic and clinical research in this field, as well as reliable and innovative treatment options for these diseases [10,11]. Primary prevention as well as secondary prevention, e.g., early detection with screening programs, is of pivotal importance to reduce the social and medical impact of these diseases. This is especially important for either highly prevalent diseases such as periodontitis (e.g., via the detection of inflammatory biomarkers in saliva), or for the detection of precancerous lesions, where the development of the actual tumor can be avoided [12,13,14,15]. In the context of OSCC’s early detection, innovative noninvasive diagnostic approaches, such as vital staining with Toluidine Blue, tissue autofluorescence, optic coherence tomography, or in vivo confocal microscopy, are promising tools since they overcome the disadvantages of classical biopsies [11,16,17,18,19].

For decades, the study of oral pathogenetic mechanisms has been a major goal of basic research in the field of oral biology. In addition to clinical studies on patients and animal models, in vitro cell culture systems have become precious tools to understand the molecular mechanisms underlying oral tissue morphogenesis as well as the above-mentioned oral diseases [20,21,22,23]. In almost all oral diseases, the cells of the periodontal tissues and the oral cavity are damaged by external noxae. These noxae consequently lead to alterations in the cells’ physiology and impair the function of the whole tissue [24]. While metabolic shifts are reversible during acute inflammation, cellular adaptations and tissue remodeling become permanent during chronic inflammation and tumorigenesis [25,26]. In the case of oral cancer, changes in the cells’ DNA via mutations and epigenome via epigenetic modifications severely impair cellular behavior [27]. Processes such as cell adhesion, migration, proliferation, differentiation, and apoptosis are dysregulated and finally lead to cellular invasion and metastasis [28,29,30,31]. In this context, cell culture platforms allow for, e.g., monitoring epithelial growth under physiological and pathological conditions, the molecular analysis of protein and gene expression patterns within the tissue, and the spatio-temporal manipulation of these processes [32]. The simulation of major pathogenetic factors, such as inflammatory stimuli or exposure to noxae such as ethanol, led to valuable insights into the pathogenesis of oral diseases [33,34]. Such a cell culture system will be presented here to illustrate the advantages but also the major shortcomings of this approach. In conjunction with clinical data, these findings are, nonetheless, the basis of the accurate diagnosis of disease entities as well as targeted, personalized treatment regimens in the future [35,36]. 

The periodontium is a complex tissue, harboring diverse cell types, including gingival keratinocytes (GKs), gingival connective tissue fibroblasts (GFs), periodontal ligament stem cells (PDLSCs), periodontal ligament fibroblasts (PDLFs), mesenchymal stem cells (MSCs), osteoblasts, and osteoclasts [37]. Although simple cell culture models form the basis of understanding in periodontal biology, they do not adequately represent the tissue architecture and are not sufficient to study tissue regeneration. This is especially important when it comes to the surgical treatment of periodontitis or OSCC, where large tissue defects need to be covered. In this context, functional tissue engineering has been an important issue in oral biology for more than a decade. In comparison with classical tissue engineering, this rather modern sub-discipline addresses both the biochemical and biophysical/biomechanical properties of a candidate biomaterial to support cell growth and tissue formation in the best possible way [38]. Compelling experimental evidence has established the concept of molecular mechanotransduction, the process by which external biophysical cues are sensed by a cell, and are subsequently transformed into intracellular biochemical signals and adaptive cellular responses [39]. Consequently, amongst others, material properties such as lack of cytotoxicity, elasticity/stiffness, and the number and density of cell adhesion points are now accepted as decisive parameters in the development of biomaterials [40,41]. The importance of environmental mechanical signals in regulating the aforementioned cell functions is evident in a paper published by the Ingber group. Herein, activation of the cellular tyrosine-kinase Sarcoma (c-Src) in response to mechanical forces is described to occur 40 times faster than that induced by the epidermal growth factor (EGF), as predicted by physical models [42,43]. This is of high relevance to the idea of the periodontium as an anatomical region with diverse tissues, which all have different biophysical properties. Thus, designing biomaterials for periodontal regeneration is extremely challenging [44,45,46,47]. A non-woven biohybrid for gingival regeneration and related work will be discussed, which illustrates the principles of biomechanics when implemented in tissue engineering and its application to oral medicine. 

Regarding periodontal disease causatives, the pathogenetic role of bacteria such as porphyromonas gingivalis (PG) [48], treponema denticola (TD) [49], tannerella forsythia (TF) [50], aggregatibacter actinomycetemcomitans (AA) [51], and others is well established. Therefore, treatment strategies for, e.g., periodontitis, can also address non-host cells, i.e., the pathogens, instead of focusing on the actual host tissue by tissue engineering. Fighting the microbes is effective in mitigating the host–pathogen interaction that contributes to the destruction of the periodontium [52,53]. Non-surgical treatment of periodontitis often includes the local or systemic administration of antibiotics such as macrolides [54]. There is still a controversy regarding the proper treatment regime, since randomized, controlled trials are scarce [55,56]. Thus, alternative intervention strategies are needed. One possibility is to stimulate the proliferation of periodontal cells by, e.g., photobiomodulation, which is especially effective for soft tissue repair [57,58]. The development of synthetic antimicrobial peptides is another promising approach to support periodontitis treatment under experimental and clinical conditions. Such peptides occur naturally or are specifically designed to kill bacteria. Many antimicrobial peptides act as pore-forming toxins or pass the cell membrane of the bacteria to interfere with the function of cytosolic proteins [59,60]. The major advantage of antimicrobial peptides is their local administration, which reduces adverse effects and antibiotic resistance [61]. Thus, such peptides are a good complement to biomaterial-based oral tissue engineering to restore periodontal function. Interesting aspects of such polymers are discussed herein.

The understanding of oral tissues that arises from cell culture studies and biomaterial/polymer-based approaches is, however, only one facet of oral regenerative medicine. Diverse other technologies and techniques have been developed, which both broaden and complement the understanding of the (patho)-physiology of the oral cavity and can be used to mitigate disease conditions. Similar to other medical disciplines, cell-based treatment strategies that make use of stem cells (SCs) derived from various periodontal tissues, but also from bone marrow, adipose tissue, or induced pluripotent stem cells (iPSCs), have become important in periodontal tissue regeneration research in recent years [62]. SCs are characterized by their developmental potential, which enables them to differentiate into diverse cell types [63]. Thus, they can theoretically be used to build up tissues or whole organs from scratch [64,65]. This property renders them an interesting tool for periodontal regeneration. A further promising approach is the intentional light-responsive control of gene expression. This research area is called optogenetics (opto = light and genetics = genetic). Optogenetic gene control makes use of proteins, whose conformation changes in response to the application of light. This conformational change induces downstream effects, which can be directed towards the intentional induction or shutdown of certain transcripts. Although initially developed for neuronal cell research, this principle can be applied to tissue engineering or tissue regeneration in dental medicine and support, e.g., cell differentiation and wound healing or suppress malignant cellular phenotypes [66,67,68,69]. Consequently, the integration of SC biology and targeted gene regulation is an auspicious extension of cell culture studies and biomaterial-based tissue engineering in oral medicine and will be referred to in this article. 

Taken together, this review is a compilation of different aspects of molecular oral research and related tissue engineering applications, which covers the evolution of experimental findings in the context of, e.g., oral carcinogenesis and periodontitis, as well as their translation into periodontal regenerative approaches in the last two decades. It does not aim to provide an in-depth discussion of one selected aspect of oral regenerative biology, but provides a broad coverage of the topic to the reader. To this end, it was decided that cell culture studies as well as sophisticated biomaterial applications should be included to draw the path from initial discoveries to later applications and to show how the different sub-disciplines interdependently and mutually influence each other. This narrative review, therefore, is an important contribution to the scientific literature since it enables easy and direct access to many facets of current oral biology research and tissue engineering approaches. The broad spectrum, ranging from molecular oral tissue homeostasis and carcinogenesis to innovative biomaterials, addresses both basic researchers and clinicians in the field of dentistry and oral and maxillofacial medicine.

## 2. Results

### 2.1. In Vitro Cell Models to Study Oral Epithelial Tissue Homeostasis and Carcinogenesis 

OSCC is a devastating disease arising from the squamous epithelia of the oral cavity. The main risk factors are alcohol ingestion as well as tobacco use or the chewing of areca nuts (especially in Asia). Infection with high-risk subtypes of human papillomavirus (HPV) is also an etiologic factor. Since symptoms occur late in the course of the disease, patients usually present with Union for International Cancer Control (UICC) stage III or IV disease [70]. Apart from radical surgery with neck dissection, radiotherapy and chemotherapy are established treatment concepts. Therapeutic antibodies such as cetuximab (directed against epidermal growth factor receptor (EGFR)) [71] or pembrolizumab, an immune checkpoint inhibitor directed against programmed cell death protein 1 (PD-1; [72,73]), expand the current treatment options, especially for advanced disease. Pharmacologically active natural compounds have also been extensively investigated for their potential antitumor activity in OSCC and may complement classical treatments in the future [74,75,76]. An interesting example is the use of extracts from propolis, a complex resinous mixture of wax, bee saliva enzymes, and plant-derived chemicals [77]. A recent study by Wezgowiec and colleagues revealed that ethanol or ethanol/hexane extracts of propolis from different regions in Poland showed remarkable anticancer effects on tongue OSCC cell lines and additional anti-inflammatory activity. The pharmacological activity is attributed to either caffeic acid phenethyl ester or a synergistic effect of different polyphenols [78]. A deeper understanding of the mechanisms of action and exact chemical composition of such natural products will, thus, prospectively enhance therapeutic options for OSCC. 

As exemplified by the use of EGFR- or PD-1-directed therapies, a molecular understanding of the pathogenesis of the disease is key to developing additional targeted treatment options. To this end, robust and reliable in vivo and in vitro disease models are needed. On the one hand, in vivo models, for instance, those based on the nude mouse system, may most adequately represent the in vivo tissue environment of an experimentally induced tumor. Such tumor models are established, e.g., via phorbol ester treatment, which serves as a tumor-promoting agent [79], or via the xenografting of cancerous cells [80]. On the other hand, in vitro models based on cell culture systems are much less complex than the in vivo models due to, for instance, restrictions on their interactions with other cell types. They are, therefore, suitable for more basic oriented molecular and biochemical studies, including drug screening. Moreover, they will help to abandon animal experiments [81]. To illustrate the advantages and limitations of such cell culture systems, in vitro cell models, in the form of conventional 2D cell cultures and 3D epithelial equivalents (EE), are discussed below.

Regarding conventional cell cultures, Gawas and colleagues reported the establishment of three cell lines derived from buccal OSCC. These cell lines express cytokeratins (K) K8 and K14 as indicators of their epithelial origin. Further, they are negative for HPV infection, exhibit aneuploidy, and form tumors in vivo [82]. Although these cell lines are good candidates for the study of OSCC in vitro, they represent the final stage within the multistep carcinogenesis process. This means that the molecular processes, which lead to proper carcinoma, have already taken place. Apart from epigenetic dysregulation, genomic instability, and dysregulated cellular energetics, this involves the enhanced, unrestrained function of several proto-oncogenes. In their constitutively active form, they are called oncogenes and lead to sustained cellular proliferation [83]. Additionally, tumor-suppressor genes that normally act as controllers of the cell functions are inactivated [84], which renders the cells resistant to apoptosis. Under in vitro conditions, this process is termed “cell transformation” and covers all changes from the “normal” to the “fully” malignant tumor cell. 

One important step during cell transformation is immortalization. This term, however, does not describe individual cells with an unlimited lifespan, but a cell population with a certain pool of permanently proliferative cells [85]. From a scientific perspective, this would optimally lead to stably cultivatable cell lines, which are suitable for research on both tissue homeostasis and cancer. The molecular basis for immortalization is the escape from cell senescence [86]. In the case of human keratinocytes, amongst others, the introduction of the E6 and E7 genes of the human papilloma virus type 16 (HPV16), either by natural infection or via transfection in vitro, can lead to immortalization. These genes abrogate cell cycle arrest by interfering with the tumor-suppressor genes and cellular gatekeepers p53 and Retinoblastoma (Rb) [87]. The E6 protein inhibits the function of p53 and leads to its degradation. This, in turn, deactivates p53’s function in growth arrest and apoptosis and promotes the cell cycle without repairing potentially damaging DNA mutations [88,89]. The E7 protein binds to Rb and leads to the dissociation of the protein from the transcription factor E2F. Consequently, E2F promotes the transition of the cell cycle from the G1 to S phase, and thereby sustains cellular proliferation [90]. The cyclin-dependent kinase (CDK) inhibitor p16 is upregulated, which can be immunohistochemically detected as an indirect marker of E7 expression in cells [91,92]. These molecular properties define the oncogenic potential of E6 and E7. Of interest, not only do the classical high-risk HPV types express these proteins in OSCC samples from patients, but low-risk types such as HPV70 also express these proteins, which can, therefore, also contribute to oral carcinogenesis [93,94].

Many cell culture OSCC models similar to that of Gawas and colleagues have been presented in the literature [95]. Their major shortcoming is that they are established from clinically apparent cancers and, therefore, do not reflect the actual process of carcinogenesis. The sequence of the molecular changes, as well as their interdependence, cannot be investigated in such models. A recent review by Suryaprakash and colleagues pointed out that scientific data on 3D cell cultures for the study of oral carcinogenesis itself are very scarce [96]. Thus, the adequate in vitro modelling of oral carcinogenesis remains a major goal in the field.

Based on this idea, a cell line derived from human gingival keratinocytes (HPV-16GM), in which immortalization was achieved by using HPV-16 E6 and E7, was established. This cell line is non-tumorigenic following nude mouse subcutaneous injection and transplantation. In the mouse model, it also lacks any signs of a benign neoplastic or invasive malignant transformation [97,98]. HPV-16GM cells exhibit all major molecular features of their native tissue, including the expression of tissue-specific K5 and K14, as well as K1 and K10, and hemidesmosomal integrin α6. The terminal differentiation markers involucrin and filaggrin, as well as epithelial €-cadherin, which is part of the adherens junctions (AJs), are also expressed. Interestingly, these molecules, which are indispensable for epithelial keratinocytes to build and maintain tissue homeostasis, could be detected in conventional monolayers, but also in in vitro generated EEs (see below) of HPV-16 GM, herein exhibiting a spatial distribution resembling the native gingival epithelial counterpart [33,98]. To check for chromosomal alterations, the status of chromosomes (Chrs) 1, 8, 10, and 18 was determined, since aberrations in these chromosomes were reported in the cell lines of aggressive head and neck cancers [99]. Regarding Chr 1, an analysis of epigenomic dysregulation in the tissue samples of patients suffering from gingival–buccal OSCC revealed a high number of differentially expressed genes. In one study, 184 of 1734 differentially regulated genes in OSCC were located on Chr 1 [100], thus emphasizing its decisive role in OSCC. In the HPV-16GM cell line, polysomy, i.e., trisomy and tetrasomy of Chr 10 and, to some extent, also Chr 18, were detectable in early passages, but these did not persist. Chr 18 monosomies were more prominent in late passages. Importantly, the relative amount of normal disomic chromosome pairs differed between the chromosomes but remained nearly constant in the early and late passages (pp. 22–83) [98]. These features render this cell line an optimal candidate for the study of oral epithelial tissue homeostasis. The latter process is defined as the lifelong maintenance of the coordinated balance between proliferation and differentiation within stratified epithelia such as skin or oral mucosa [101,102]. It can serve as a good experimental control in the study of progressive stages of cell transformation and oral carcinogenesis [103]. 

Regarding oral carcinogenesis, in addition to infection with high-risk papilloma viruses such as HPV-16 and HPV-18, lifestyle causatives such as areca nut, tobacco and alcohol consumption are well-accepted risk factors. There is, however, growing evidence that OSCC can also be caused by chronic mechanical irritation (CMI) [104]. CMI may arise through dental prostheses, poor dentition, or behavioral factors such as cheek biting [105]. The molecular mechanisms of these carcinogens have been intensively investigated. While components of areca nuts such as arecoline interfere with different DNA repair mechanisms [106,107], the molecular basis of CMI-related carcinogenesis is still an open research question. 

For tobacco-associated OSCC, an Indian study reported frequent mutations in molecules, such as the tumor-suppressor p53 and the small GTPase Harvey rat sarcoma virus, p21^ras^ (H-ras) [108]. A recent study conducted with immortalized oral keratinocytes also found changes in the expression of the tumor-suppressing cell–cell-contact molecule E-cadherin. Loss of E-cadherin is seen in many carcinomas, including OSCC [109]. It is associated with epithelial–mesenchymal transition (EMT, see below), and a migratory, invasive cellular phenotype [110]. Additionally, several microRNAs were proposed to be involved in oral carcinogenesis, since the differential or neo-expression of these regulating RNAs was reported in response to chewing tobacco and smoke exposure [111,112]. 

In the case of alcohol consumption, the main component, ethanol, is oxidized to acetate in two steps. First, alcohol dehydrogenases convert ethanol to acetaldehyde (HAc). Second, HAc is oxidized to acetate via aldehyde dehydrogenases. HAc damages cells by covalently modifying proteins, including histones, and by interfering with the important cellular glutathione antioxidant system. Lipids and DNA are covalently modified by HAc through adduct formation [113]. Further, this leads to the synthesis of toxic cell metabolites such as salsolinol, which induce reactive oxygen species (ROS) formation, further damaging the cell [114]. In this context, it could be shown that the exposure of normal oral keratinocytes to Hac promotes malignant transformation. Mechanistically, the methylation status of various genes, including the erbB2 gene, is altered. erbB2 is also known as HER2/neu, encodes for the receptor tyrosine kinase erbB-2, and is amplified in many cancer entities. The study by Miyazaki and colleagues could show a HAc-induced demethylation in the erbB2 gene, which leads to an increased expression of the gene, thereby sustaining cellular proliferation and contributing to oral carcinogenesis [115]. 

Due to the decisive role of ethanol in the pathogenesis of oral cancer, the HPV-16GM (“wild-type” GKs) cells were subjected to long-term ethanol exposure to simulate chronic alcohol ingestion as a risk factor in OSCC. Under these experimental conditions, the nine-week administration of 30 mM ethanol induced two stable phenotypes that were different from the “wild-type” GKs: an epithelium-like/epitheloid cobblestone morphology (EPI) and a spindle-shape fibroblast-like/fibroblastoid morphology (FIB). The cells were subsequently analyzed for the phenotypic and molecular changes emerging from this treatment.

On the molecular level, FIB cells exhibit a loss of K14 and desmoplakins, which are characteristic marker proteins of cells derived from stratified epithelia. Of interest, neo-expression of the simple epithelial keratin K18 and the mesenchymal intermediate filament vimentin could be detected. Altogether, these molecular changes are typical of EMT, the stepwise transformation of epithelial cells into mesenchymal cells during the multistep process of carcinogenesis. These data suggest that FIB cells represent a stable intermediate within this process and are, therefore, a more transformed phenotype than EPI cells [116]. Further molecular analysis revealed that the gp91*phox* homolog Nox1, an ROS-synthesizing nicotinamide adenine dinucleotide phosphate (NADPH) oxidase, is upregulated in FIB cells. Therefore, Nox1 is predominantly localized in cell membranes and nuclei. Thus, FIB-inherent Nox1 appears to be involved in the acquirement of a more advanced stage of transformation of the FIB phenotype. This suggestion is underlined by detection of three important ROS species, namely, HOO, H_2_O_2_, and OH, in membrane fractions of FIB cells, and by upregulation of Nox1 downstream targets c-Jun-N-terminal kinase 1 (JNK1) and Mitogen activated kinases p44/42 (ERK1/2), as well as increased phosphorylation levels of JNK2 [97]. The latter molecules are all part of the mitogen-activated protein kinase (MAPK) pathways, contribute to proliferation and cell survival, and are thus of great importance to carcinogenesis [117]. In the ERK1/2 context, further mechanistic studies employing the ERK1/2 inhibitor UO126 demonstrated that activated states of p44/42 in FIB cells are responsible for elevated inducible nitric oxide synthetase (iNOS) at both the mRNA and protein levels. This suggests that these kinases function as iNOS upstream regulators [118]. iNOS is, for example, important for the activation and migration of macrophages [119]. However, it has attracted increasing attention in cancer research since it is highly expressed in multiple human cancer entities, including OSCC. In OSCC, nitric oxide (NO) production by iNOS contributes to angiogenesis, tumor-suppressor inactivation, and the invasive phenotype. Although not statistically significant, iNOS expression increases from normal oral mucosa to precancer histologic lesions and the fully malignant OSCC phenotype [120]. The abundance of the NO-dependent nitrotyrosine modification of proteins is also a prognostic marker in OSCC, meaning that higher nitrotyrosine levels are associated with a poor outcome [121]. In addition to influencing iNOS, the above-mentioned intervention on ERK1/2 suppresses the anchorage-independent growth (AIG) of FIB cells and reduces vimentin expression [118]. AIG reflects the ability of in vitro transformed cells and cancer-derived cells to survive and grow in the absence of anchorage to the extracellular matrix (ECM) and their neighboring cells. AIG is a hallmark of carcinogenesis, and evaluating its degree has been used in the analysis of epithelial cells at different transformation stages in a semisolid medium called soft agar, where the degree of cell transformation is reflected by the formation of cell colonies [122]. The anti-tumor activity of pharmacological compounds can also be evaluated via testing the AIG of cells. In the context of OSCC, 6-shogaol, an ingredient in dried ginger, has exemplarily been shown to reduce AIG of OSCC cell lines, meaning that the compound inhibits the cancerous properties of the cells and might also be used as a complementary cancer treatment [123]. 

To analyze further molecular alterations in FIB cells under more in vivo-like conditions, organotypic cocultures were established. These are characterized by the spatially separated growth of mesenchymal fibroblasts and epithelial keratinocytes. These cocultures allow for keratinocytes to form stratified 3D-epithelia under in vitro conditions, which are also termed EEs [98,102]. Compared to “wild-type” GKs and EPI cells, FIB cells are less able to form proper epithelia and exhibit a striking down-regulation of epithelial-specific K14, hemidesmosomal α6 integrin, and the AJ constituent E-cadherin [33]. The progressive replacement of epithelial E-cadherin with mesenchymal N-cadherin, termed a cadherin switch, has recently been shown to be critical for OSCC tumor progression, in both a mouse model and human OSCC samples [124]. Similar to the monolayer culture, FIB-derived EEs show extensive vimentin abundance [33]. This switch to mesenchymal cell markers is not only indicative of EMT, but can also be found at the invasion front of, for example, grade IVC OSCC tissue samples, as demonstrated by Kato and coworkers [125]. These findings underscore the intimate interrelationship between E-cadherin, vimentin, EMT, and the invasive/malignant cell phenotype. A recent study from Thailand on 200 patients suffering from OSCC revealed that the analysis of combined E-cadherin and vimentin expression as an indicator of EMT has a superior prognostic significance when compared to the individual protein expression levels. The authors concluded that the aggressiveness of the tumor is strongly correlated with the degree of EMT, i.e., tumors with complete EMT are more aggressive than those with only partial EMT [126]. 

The functional consequences of the E-cadherin downregulation in FIB cells were further analyzed. It has been described in the literature that E-cadherin downregulation is associated with a poor outcome and an aggressive histologic phenotype in OSCC. Molecularly, E-cadherin downregulation is established either via promotor methylation, RNA downregulation or the increased co-internalization of the protein with EGF and EGFR [127,128]. Of interest, the cadherin family member placental (P)-cadherin is also a prognostic OSCC marker. The expression of P-cadherin, especially when localized at the plasma membrane, indicates better survival [129,130]. 

Thus, the expression and co-localization of E-cadherin and its intracellular AJ binding partner β-catenin were studied at the plasma membrane in the EPI and FIB phenotypes. If E-cadherin and β-catenin are found in proximity, this is indicative of intact AJs. Using stimulated emission depletion (STED) microscopy, it could be shown that EPI cells exhibit the membrane co-localization of both proteins, whereas FIB cell do not. This indicates a disruption of AJs in the FIB cells, which has consequences for epithelial integrity [103]. The persistence of β-catenin with a concomitant downregulation of E-cadherin was also reported in fully malignant OSCC, while the highest proportion of β-catenin was found in poorly differentiated OSCC (PDOSCC) [131]. In this context, it should be noted that, in PDOSCC, most of the β-catenin was detected within the cytoplasm [131]. It is, therefore, likely that β-catenin also acts as a transcription factor in this case, and promotes cell proliferation due to its nucleo-cytoplasmic shuttling ability. The nuclear importation of β-catenin, which is indispensable for its transcription factor and proliferative activity, could be shown in vitro to be mediated by the importin family member importin-11/IPO11, which shuttles cargo from the cytoplasm to the nucleus [132]. A comparative study on keratinocytes obtained from dysplastic and non-dysplastic oral tissue revealed the stabilization and accumulation of β-catenin in the nucleus of dysplastic keratinocytes. The endocytic protein Rab5, which is required for endosomal sequestration of the β-catenin destruction complex, was involved in this process. Moreover, the authors of this study could correlate the in vitro results with in vivo data. In clinical samples of oral dysplasia, they showed an increased co-localization of the β-catenin destruction complex constituents glycogen synthase kinase 3 beta (GSK3β), adenomatous polyposis coli (APC), and the scaffold protein axin, with early endosome antigen 1 and Rab5-positive early endosomes [133]. Taken together, these data suggest the division of the E-cadherin and β-catenin functions is a key driver of oral carcinogenesis. β-catenin’s switch from adhesion to its transcription factor function, as well as its indirect stabilization through reduced degradation, seem to be crucial for EMT and to maintain proliferation and oncogenic signaling in the transformation of oral keratinocytes. 

Another interesting facet of the FIB phenotype arises from the reduced abundance of the protein zyxin in fractions obtained from FIB plasma membranes, while large proportions can be found within cytoplasmic protein fractions [103]. Generally, zyxin binds to actin stress fibers and focal adhesions (FAs). It is also present in AJs to facilitate cytoskeletal reinforcement in response to mechanical stress [134]. Under conventional cell culture conditions, the rigid polystyrene material exerts a great mechanical stress on cells. This extracellular mechanical stress is sensed by both cell–matrix adhesion structures, i.e., FAs, and cell–cell contacts, i.e., AJs. Extensive crosstalk between FA-innate integrins and AJ-inherent cadherins leads to the coordinated fine-tuning of cellular mechanotransduction [135]. Thus, it cannot be ruled out that the disruption of AJs in FIB leads to a shift from membrane-bound to cytoplasmic zyxin. One of the rare mechanistic studies revealed that, in breast cancer cells, cytoplasmic zyxin can inhibit the HIPPO pathway, thereby promoting cell proliferation via nuclear translocation of the proliferation-associated co-transcriptional activator yes-associated protein (YAP) [136]. Regarding OSCC, it has been reported that silencing of the zyxin gene in OSCC-derived cells prevents the promotion of proliferation, migration, and invasiveness [137]. These reports provide evidence that zyxin, in addition to being involved in cell-to-cell and cell–matrix adhesions, is also involved in other features of cell behavior. YAP and its cellular homologue, the transcriptional co-activator with PDZ motif (TAZ), both bind to TEA-domain (TEAD) transcription factors, particularly TEAD2, to exert their biological function inside the nucleus. In consecutive mechanistic experiments, employing a pharmacologically based approach, it could shown, for the first time in periodontal cells, that YAP, rather than TAZ, governs the expression of zyxin, which appears to be a YAP target gene [134]. These mechanistic insights broaden the understanding of cellular adhesion structures and mechanosignaling pathways in the context of periodontal cells and OSCC development. 

**Figure 1:**
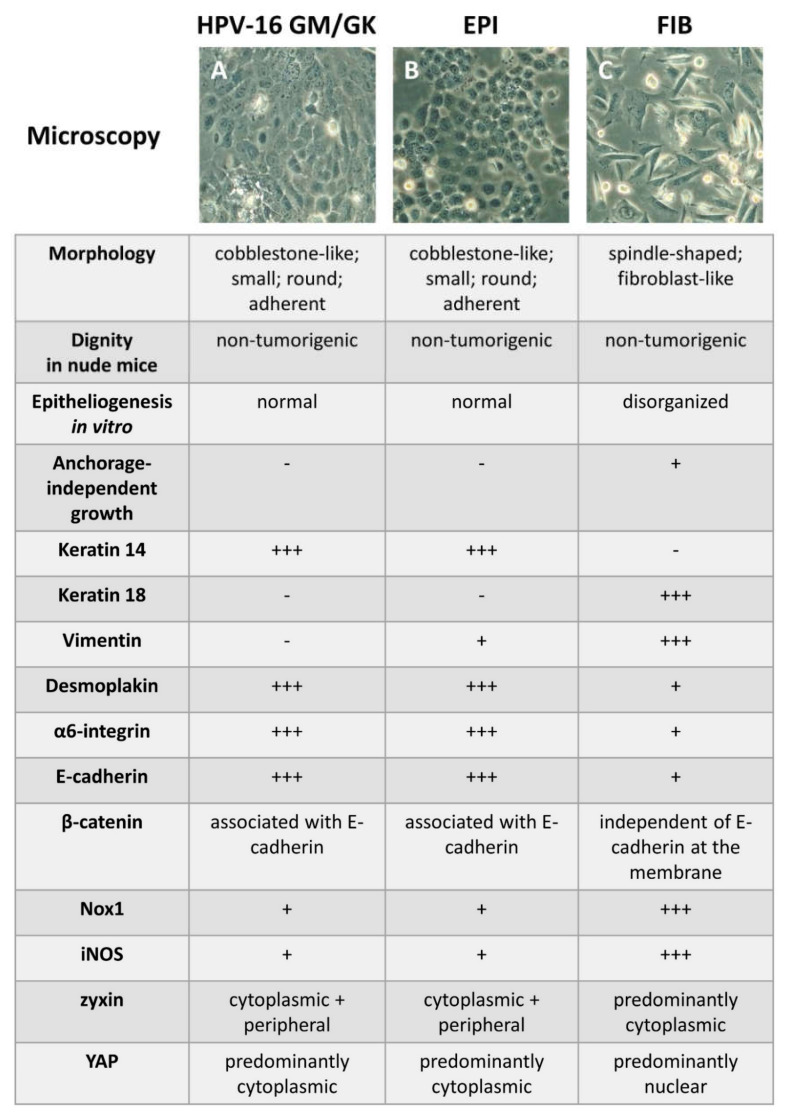
Summary of the morphological and molecular properties of HPV-16 GM (=GK), and the alcohol-treated cell lines EPI (epitheloid) and FIB (fibro-blastoid). The upper part of the figure depicts exemplary light microscopy pictures of the cell lines at the same magnification. (**A**) shows the GKs with their cobblestone morphology, which is typical for keratinocytes. (**B**) depicts EPI cells, which share morphological similarities with GKs but show more cytoplasmic inclusions and more pronounced nuclei. In (**C**), the fibroblast-like, spindle-shaped FIB cells are presented. When compared to GKs and EPI, FIB cells do not resemble normal keratinocytes and are characterized by multidirectional cytoplasmic extensions and a less dense growth pattern. The lower part of the figure summarizes the main cell behavioral and molecular properties of the three cell lines. The protein expression levels are designated as follows: +++ very strong expression, + strong expression, - no expression. Details are given in the main text. (Nox1 = NADPH Oxidase 1; iNOS = inducible Nitric Oxide Synthase; YAP = Yes-Associated Protein.)

At the cell behavioral level, FIB exhibited a much faster response to proliferation-promoting fetal calf serum, and thus required a much shorter timespan for cell doubling compared to EPI and the parental HPV-16GM cells. FIB cells also exhibited a striking nuclear YAP localization and higher amounts of chromatin-bound YAP when compared to EPI and HPV-16GM. These results, therefore, demonstrate that phenotypic differences in alcohol-treated keratinocytes correlate with discriminative proliferation behavior. Two molecular equivalents of this cell behavior are the increased cytoplasmic fraction of zyxin and a higher nuclear YAP abundance. Furthermore, the discussed molecular and cell behavioral differences indicate that these alterations in FIB represent very early changes in epithelial cell transformation during alcohol-induced oral carcinogenesis [103]. Functional studies on OSCC cells, as well as an immunohistochemical investigation of patient samples from OSCC, also describe the involvement of YAP [138,139,140]. The upstream regulators of this YAP-dependent proliferation are, however, diverse. Ion channels such as PIEZO1- or G-protein-coupled receptors (GPCR) such as GPR39 have been described in the literature [139,140]. It is very likely that the very stiff ECM found in many carcinomas, also called desmoplastic stroma, is an indirect regulator of nuclear YAP activity and, therefore, links mechanotransduction to cellular proliferation [141]. The morphological and molecular changes of FIB cells are compared to EPI and HPV-16GM cells and graphically summarized in Figure 1.

In conclusion, the aspects of alcohol-induced oral carcinogenesis and cell transformation discussed in this section show that many molecular changes detected in clinically apparent oral carcinomas, such as the loss of E-cadherin, gains in vimentin, or the nuclear abundance of YAP, can be demonstrated with the presented in vitro cell model. Since the cells are transformed but do not yet show any tumorigenicity in vivo, the observed molecular changes represent very early stages of carcinogenesis. To date, to the best of our knowledge, no other stable cell models are available that reproducibly reflect these cell transformation events within the multistep process of oral carcinogenesis. Previous models are mostly derived from already established OSCCs, or are not sufficiently molecularly and genotypically characterized [82]. Consequently, the model cell lines, (i) HPV-16 GM as a parental cell line, (ii) EPI, and (iii) FIB, are a valuable investigation platform to identify OSCC-relevant molecular alterations at very early stages of oral carcinogenesis, and to characterize their effects on cell behavior. This renders this kind of in vitro model as an optimal candidate for translational research on molecular tumor diagnostics and targeted therapies for OSCC. The molecular characterization of oral cell lines under physiological and pathological conditions deepens the understanding of oral biology. This knowledge forms an indispensable basis for regenerative treatment approaches in the oral cavity, which are discussed in the following section. 

### 2.2. Biomaterial-Based Approaches in the Context of Oral Diseases

The biomaterial-based regeneration of oral tissues is a complex issue, since the periodontium is a highly specialized anatomic compartment with both soft and hard tissues. Material selection is, therefore, an essential first step in establishing sustainable and successful treatment options [142]. Important parameters include biocompatibility, biodegradability, and mechanical strength [143]. The choice between natural or synthetic polymers or hybrid approaches determines the physicochemical properties of the scaffolds. Next, the fabrication mode, e.g., electrospinning, 3D printing, or salt leaching, contributes to the material properties. Functionalization of the scaffold with chemically active residues (e.g., coating with ECM components) influences the material’s interaction with different cell types, which is the reason that an in-depth cell biological characterization of the target cells is crucial (see above) [142,144].

Currently, guided tissue regeneration, which prevents epithelial overgrowth in periodontitis treatment, autogenous bone substitutes, and diverse natural and synthetic polymers (e.g., polylactic acid, polycaprolactone, chitosan, alginate, collagen, silk) are in clinical use in oral medicine. The advantages and shortcoming of these approaches are discussed elsewhere [142,144]. Functional tissue engineering, however, also considers the mechanobiological aspects of the target tissue. Therefore, examples of biomaterial design for oral epithelial and soft tissue regeneration are discussed next [145].

#### 2.2.1. Nonwoven Biomaterial Design for Oral Epithelial and Soft Tissue Regeneration

In the oral cavity, shear stress is ubiquitous [146,147]. Therefore, biomaterials being developed for translational research and future clinical application must consider this crucial aspect. This is important, e.g., in the context of epithelial and soft tissue regeneration (STR) required for oral cavity diseases such as carcinomas or for profound soft tissue loss or damage due to trauma [148,149]. Another critical aspect in biomaterials research with the purpose of STR is that they must consider the loss of volume in the addressed tissue during the long-term process of wound healing [150]. These two aspects make it essential that the device for STR in the oral cavity is elastic enough to withstand multidirectional shear forces. It should also remain at the wound site for a sufficiently long time to allow for compensation of the volume loss. Regarding the clinical use of biomaterials in the oral cavity, it should also be noted that they must be easy to handle for the surgeon, who ultimately performs the wound treatment. From our own experience, one criterion for good handling is the strength of the material, which must be sufficiently high that it does not tear when sutured into the tissue defect. 

**Figure 2:**
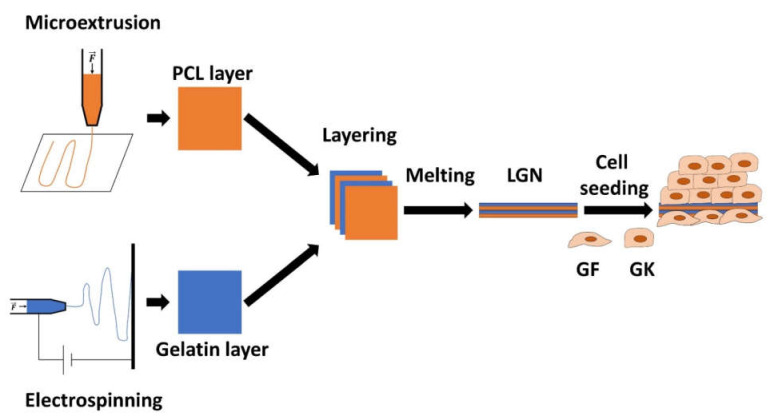
Schematic representation of the manufacturing process of the layered gradient nonwoven (LGN). The polycaprolactone (PCL) layers were generated by 3D micro-extrusion (upper left part; orange). During this process, the polymer solution is extruded through a narrow orifice and deposited on a carrier platform. The gelatin layers were generated by electrospinning (lower left part; blue). The polymer solution is also extruded through an orifice but is additionally accelerated towards a collector (black bar) via an electric field. Alternating layers of PCL and gelatin were then briefly heated above the melting point of PCL, yielding the LGN. Subsequently, the cells (GF = gingival fibroblast; GK = gingival keratinocyte) were transferred on opposite sides of the LGN and incubated in culture medium. The resulting stratified epithelium and the layer of GFs is depicted on the right side. Details are given in the main text. ($\vec{F}$ = mechanical force).

Considering these requirements, a biomaterial was developed as a scaffold for either in vitro cell culture or in vivo application. The aim was to allow for the growth of primary or immortalized oral cells in vitro, including proper epithelial stratification. For in vivo use, colonization by tissue resident cells from the in situ defect site should be achieved. A biohybrid, polymer-based biomaterial was used. This was configured as a layered gradient nonwoven (LGN) through electrospinning and is composed of natural collagenous gelatin nanofibers and the biodegradable synthetic polymer polycaprolactone (PCL), the latter produced by 3D micro-extrusion. This device aims to form a three-dimensional template that mimics the ECM of soft tissues for proper cell adhesion and growth. Concerning gelatin, the use of a non-toxic solvent/cross-linker mixture comprising acetic acid, ethyl acetate, water and glyoxal facilitated the in situ crosslinking of gelatin nanofibers with varying diameters (90–680 nm) during electrospinning. This increases water resistance and prevents post-treatment crosslinking damage to the gradient gelatin network. Notably, the cohesion between the electrospun gelatin and extruded PCL was ensured by a lamination process in which the PCL was briefly heated above its melting temperature. Such sandwich-like devices have been proven to be compatible with both gingival keratinocytes and connective tissue fibroblasts. Furthermore, the in situ biodegradation of the LGN varies with the number of laminated gelatin-PCL layers, so that the half-life of the biomaterial at the defect site can be adapted to the specific need. This helps to overcome the obstacles associated with the aforementioned loss of volume in the tissue [151]. The manufacturing process of the LGN is depicted in Figure 2. 

We then aimed to evaluate the suitability of the LGN for clinical application using pre-clinical in vitro and in vivo validation studies. Primary GFs and immortalized GKs (HPV-16GM, see Section 2.1) could populate the whole LGN. Investigations on tissue-specific intermediate filaments revealed vimentin expression in connective tissue fibroblasts. HPV-16GM cells expressed K14 as well as K1/10, with the latter indicating early keratinocyte differentiation. The ability of HPV-16GM cells to express involucrin and filaggrin on the LGN demonstrates that growth on this biomaterial allows for regular keratinocyte differentiation [152], since involucrin and filaggrin represent terminal differentiation markers [102]. The LGN also enables the guided formation of stratified EEs in vitro when fibroblasts of the gingival connective tissue and HPV-16GM keratinocytes are established as cocultures. This led to a proof-of-concept study in a minipig model. After inducing soft tissue dehiscence, a nearly complete wound closure was observed at three weeks post-surgery, as indicated by connective tissue formation and re-epithelialization. Importantly, failure of wound closure was observed in cases of LGN loss due to mastication, while at sites with the presence of LGN, the tissue was devoid of any signs of inflammation throughout the entire observation period. These findings confirm both the biocompatibility of the biomaterial and its potential clinical applicability [152]. 

During regenerative processes such as wound healing, the synthesis of fresh ECM molecules from wound fibroblasts, in conjunction with their assembly into an intact ECM within the wound bed, is indispensable. This is also a prerequisite for consecutive wound closure through keratinocytes, which centripetally migrate from the wound margins into the new ECM [153]. Hence, a precondition for tissue regeneration is the assembly of the molecular constituents of the ECM, e.g., collagen type-IV and laminin-1/10, into a tissue-specific three-dimensional network to provide the tissue-inherent biomechanical properties needed for tissue regeneration. Based on their molecular constituents and their architecture, each ECM offers a distinct set and spacing of anchor points, such as the fibronectin (FN)-innate arginine-glycine-aspartate (RGD) sequence for cell adhesion, and a certain stiffness, termed the Young’s modulus [154,155]. These two biomechanical parameters are essential criteria for the cells’adhesion to the ECM via, for instance, FAs integrins to perceive the ECM’s Young’s modulus. During the perception of the integrin-mediated ECM stiffness, cells convert the extracellular biomechanical signals into intracellular biochemical signals, which, when transmitted into the nucleus, govern gene expression and cell behavioral responses such as proliferation or differentiation [156]. This conversion of extracellular (bio)mechanical signals into intracellular biochemical signals at the plasma membrane is called mechanotransduction. Due to this functional significance for cell fate, mechanotransduction is pivotal for tissue regeneration and tissue homeostasis [157,158].

Regarding the biomechanically relevant spacing of cell adhesion points, polymer-based model surfaces with a defined spacing are precious investigation tools. With such substrates, favorable and unfavorable anchor point distances can be determined for different cell types. Therefore, microposts/pillars made of FN-biofunctionalized polydimethylsiloxane (PDMS) were designed. By using different distances between the posts, it was shown that the early differentiation of GKs, which is a prerequisite for epithelial regeneration, is governed by inter-post distances. K1, an early differentiation marker of keratinized epithelia, such as skin or attached gingiva, was highly expressed, with post distances of 8 µm. In contrast, larger micropatterns of 11 and 14 µm led to progressive perinuclear K1 filament condensation, along with decreased K1 gene expression levels [159]. Further studies, including other periodontal cells such as GFs, PDLFs, and alveolar bone cells (ABCs), revealed that defined variations in PDMS posts micropatterns could distinguish critical post distances in the context of periodontal tissue homeostasis. These distances are decisive for (i) GFs to support homeostatic EEs formation and biomarker expression in GF-HPV-16GM cocultures, and for (ii) PDLFs and ABCs to express homeostasis-relevant marker proteins. In PDFLs, collagen type I synthesis is important, which is a main component of the tooth anchoring Sharpey fibers. In ABCs, alkaline phosphatase expression was monitored, since this is relevant to bone formation [160]. These PDMS–based extracellular model environments provide further evidence for the decisive role of the spacing of anchor points for cell adhesion in periodontal cell and tissue functions, such as a broader keratinocyte differentiation, epithelial stratification, and periodontal homeostasis. 

Concerning substrate stiffness, a variation in the Young’s modulus of the above-described LGN led to valuable insights into mechanically induced epithelial morphogenesis. Typically, connective tissue fibroblasts are needed to induce epithelial stratification for in vitro coculture systems, which traditionally employ collagen type-I lattices as the cell-carrying matrix [161,162]. However, it was found that LGNs with a stiffness of 3.2 kPa (determined by Bioindenter™ measurements) allow for an in vivo epithelial-like tissue morphogenesis in the absence of a connective tissue fibroblast, while the epithelial morphogenesis on softer (2.1 ± 1.0 kPa) or harder (10.9 ± 2.51 kPa) substrates was less satisfactory [163]. This morphogenesis was substantiated by keratinocyte stratification as well as the expression and proper spatial distribution of specific biomarkers, which are indicative for tissue homeostasis. The EEs display a supra-basal expression of the early differentiation marker K1 and the terminal differentiation marker involucrin. The basement membrane component collagen type IV is expressed at the epithelium-LGN interface, resembling the normal in vivo situation. This pre-clinical model of LGN-induced epithelial morphogenesis illustrates that its innate biomechanical information, represented by the 3.2 kPa Young’s modulus, can support the growth of an epithelium with proper biomarker expression in the absence of cocultured fibroblasts. Notably, the LGN Young’s modulus of 3.2 kPa is in the stiffness range of natural epithelial basement membranes of the skin or the cornea (2.0–10.0 kPa) [163]. 

To test whether the 3.2 kPa LGN is also able to support fibroblast-independent epithelial morphogenesis in vivo, constructs of GKs precultured for one day on the LGN without and in combination with GFs were transplanted into subcutaneous pockets of nude mice. The studies aimed to test the influence of a complete surrounding mesenchyme (representing the in vivo situation) or, additionally, in vitro cocultured (precultured) GFs on epithelial morphogenesis. The results of these experiments have shown that both a complete mesenchyme in vivo and the presence of precultured GFs in vitro did not lead to any improvement in epithelial morphogenesis compared to in vitro constructs consisting of GKs and LGNs only. These findings suggest that the existence of other cell entities is not required for LGN-induced GK epithelial morphogenesis in vivo and that the LGN’s epithelium-inducing effect can, therefore, be generalized. Skin keratinocytes (epidermal keratinocytes = EKs) were also tested on the 3.2 kPa LGN to evaluate whether the Young’s modulus of this LGN is also qualified to support epidermal epithelia. Here, differences were found between solitarily transplanted EKs on the LGN into the nude mice and those EKs with additional precultured epidermal fibroblasts (EFs). Of interest, the presence of EFs improved the spatial expression patterns of differentiation biomarkers such as K1 and involucrin [164]. This implies that, based on its biomechanical/biophysical properties alone, the LGN is not able to finetune differentiation for EKs. Furthermore, these findings support the notion that, in the context of tissue regeneration, there is a specific elastic modulus for each keratinocyte entity, which can support the morphogenesis of the respective epithelial type in the best possible way.

The strong relationship between molecular studies and biomaterial-based experiments is underscored by further mechanistic studies with EEs established on the 3.2 kPa LGNs. They revealed that a signaling axis consisting of the molecules’ EGF receptor (EGFR), the MAPK ERK1/2 (p44/42), and integrin β1 is strongly involved in the in vivo-like epithelial morphogenesis [165]. During epithelial morphogenesis, the LGN-innate stiffness must be transported into the GKs. To identify the key molecules involved in this mechanosensing and mechanotransducing process, an RNA interference (RNAi) approach was used to achieve an FAK shutdown. The latter kinase is, amongst others, involved in the establishment of FAs. In comparison with non-treated controls, GK-derived EEs with FAK shutdown were characterized by a dysbalanced morphogenesis, as indicated by epithelial hyperplasia and dysregulated proliferation and differentiation. This dysregulation became detectable by the persistence of keratinocyte proliferation and premature spatiotemporal abundance of K1, as well as involucrin and filaggrin [102]. These findings demonstrate the essential role of FAK within the mechanotransduction-related gingival epithelial morphogenesis and prove its importance for the biomaterial-based tissue regeneration of oral epithelia in the course of oral diseases.

The concept of using gelatin-based nonwovens for periodontal tissue engineering was further refined in recent in vitro/in vivo studies. It should be noted that nutrient supply via proper vasculature/angiogenesis is a critical issue in tissue regeneration. Taking this into account, it could be shown that the in situ crosslinking of electrospun gelatin in conjunction with a gelatin fiber diameter of 1000 nm significantly supports (i) the population of the nonwovens with endothelial cells and (ii) their angiogenic biomarker expression (vascular endothelial growth factor (VEGF), angiopoietin-1 (Ang1) and integrin β1), both in vitro and in vivo [166]. For comparison, a fiber diameter ranging from 400 to 700 nm yielded the LGN, which excellently supported the gingival epithelial morphogenesis [163]. These findings strongly suggest that slight modifications to the gelatin-based nonwovens pave the way for further applications within the field of guided tissue regeneration. This conclusion is supported by a study by our group (manuscript in preparation). Hydroxyapatite-enhanced (imitating the mineralized matrices of the periodontium) gelatin nonwovens were fabricated via electrospinning, either with or without additional pores. The latter were incorporated into the material using extractable polyethylene glycol fibers. The nonwovens were subsequently colonized with human MSCs (hMSCs), PDLFs, or cocultures of both cell types. Cell viability and differentiation was assessed by Resazurin staining and the immunohistochemistry of the differentiation marker proteins periostin, osteopontin, and Oct4, respectively. The study could show the overall biocompatibility of the material. Of interest, nonwovens with additional pores were more favorable for cell adhesion and survival. Osteopontin and periostin expression were most detected in hMSCs and PDLFs cocultures on the nonwovens. Oct4 expression was, as expected, limited to hMSCs. These data clearly show that such composite materials are (i) promising for periodontal regeneration, (ii) support the survival and proliferation of various periodontal cell entities, and (iii) lead to the differential expression of key differentiation markers in the respective cell types. Future refinement of this approach and its combination with materials that support oral mucosal regeneration (see above) may, therefore, be suitable to reproduce the complex histological and cellular architecture of the periodontium and enable its proper regeneration.

Furthermore, current trends in nonwoven design focus on functional fiber generation obtained by nonwoven post-modifications or pre-treatments. Hence, previously known biocompatible polymers, i.e., the biodegradable poly(lactide-co-glycolic acid) (PLGA), have found widespread use in modern medical practice and their degradation rate was modified by electron beam irradiation [167]. The molecular weight of PLGA nanofibers and the mechanical stiffness of the PLGA nanofibrous mats decreases with increasing electron beam radiation doses. Cell proliferation on all electron-beam-irradiated PLGA mats remains unaffected [167]. 

In the context of current orthodontic and orthopedic practice, Norkin and coworkers analyzed the therapeutic potential of modified PCL scaffolds coated with vaterite for bone regeneration [168]. Vaterite is a metastable polymorphic modification of CaCO_3_, occurring in spherical hexagonal polycrystalline microparticles with a size ranging from a few hundred nanometers to several micrometers. This modification was shown to be applicable as an osteoconductive material [169,170], which is able to effectively release Ca^2+^ ions. This is of vital importance for the osteogenic activity of osteoblasts [168]. 

Silk has also been used in biomedical applications due to its various sources, good biocompatibility [171], and tunable mechanical properties [123]. At present, silk research for tissue regeneration demonstrated new preparation techniques, transforming natural silk nonwovens into bio-adhesives for tissue repair, called artificial silk nonwovens. Here, the technique entailed pretreatment of the silkworm cocoon sheet (SCS) with a CaCl_2_-ethanol-H_2_O ternary solution to obtain a modified cocoon sheet (MCS), followed by surface modification with a CaCl_2_-formic acid (Ca-FA) solution to obtain MCS/Ca with controllable adhesion, which was achieved by adjusting the Ca^2+^ content in Ca-FA. The highly stretchable MCS/Ca firmly adhered to various substrates for loads as high as 54 kPa, and its performance in repairing an injured liver in vivo was superior to that of a commercial product, Sorbalgon^®^ [172]. 

Among the materials that are still of interest to the many scientific teams working on tissue regenerative biomaterial solutions, carbon fibers and fibrous structures based on these are the focus in the production of carbon-derived nonwovens [173]. However, because carbon fibers generally have a low biocompatibility, there is a strong need for biological functionalization to improve cell compatibility. In this context, Frączyk’s group investigated the possibility of chemically modifying the surface of carbon nonwovens made of micrometer-sized carbon fibers, using a method based on the in situ formation of diazonium salts of aromatic amine derivatives [174]. The advantage of this approach is the preparation of modified materials in which the modifying agent is bound to the surface via a stable C–C bond. The possibility of using this method of functionalization allows for the incorporation of derivatives containing various functional groups, enabling the further functionalization of carbon nonwovens, e.g., with biologically active RGD-type peptides [174]. 

These examples clearly show that the trend in the development of multifunctional biomaterials for oral regeneration is moving towards the mimicry of extracellular matrix properties using polymer-derived nonwovens and combinatorial surface modifications. These approaches, for example, exploit topographical features or variations in mechanics combined with biochemical cues. These multifunctional biomaterial approaches not only enable tissue regeneration, but also inversely stimulate the research on key regulatory characteristics regarding cell fate decisions and histogenesis. These latter aspects have recently been reviewed in the context of multifunctional biomaterial design [175].

#### 2.2.2. A Smart Hydrogel for Controlled Drug Release in Periodontal Regeneration 

**Figure 3:**
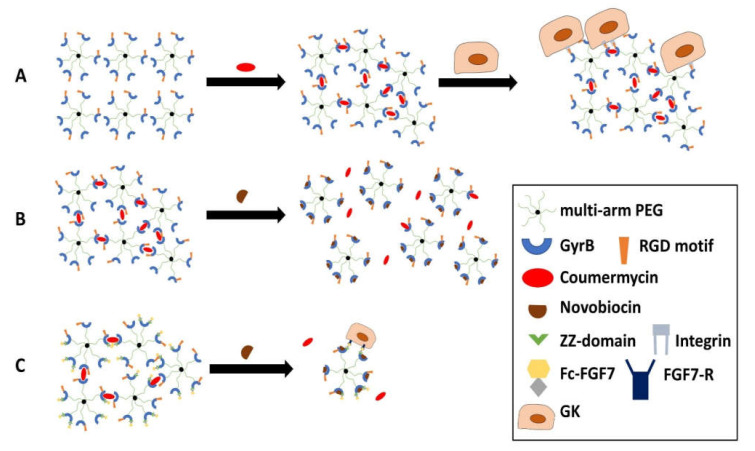
Illustration of the polyethylene glycol (PEG)-based, pharmacologically tunable “smart” hydrogel. (**A**) Multi-arm PEG (green asterisk-like structure) was used as a backbone for the hydrogel. It was chemically coupled to Gyrase B (GyrB; blue hemicycle) and the arginine–glycine–aspartate binding motif (RGD motif; orange bar) from fibronectin. Upon the addition of coumermycin (red ellipse), GyrB molecules can dimerize, which leads to hydrogel assembly. Subsequently, cells (as exemplarily represented by gingiva keratinocytes (GKs)) can be seeded on the hydrogel, which can bind to the RGD motif via membrane-inherent integrin receptors (grey receptors). (**B**) Dissociation of the hydrogel is induced by adding novobiocin (brown hemicycle). Novobiocin competes with coumermycin for the GyrB binding sites. Contrary to coumermycin, novobiocin does not lead to dimerization of the GyrB molecules. Excess amounts of novobiocin, therefore, lead to the disassembly of the hydrogel. (**C**) When additionally coupled with the ZZ-domain of Protein A (green triangle), the multi-arm PEG can bind fibroblast growth factor 7 (FGF7), which is modified with Fc (crystallizable fragment of antibodies; yellow/grey construct). When adding novobiocin, the hydrogel disassembles and FGF7 can then bind to FGF7 receptors (FGF7-R; dark blue receptor) on cells such as GKs. Details are given in the main text. A graphic legend is included in the lower right corner.

Although nonwovens offer great potential for biomaterial-based regeneration in the oral cavity, other types of materials, such as hydrogels, also exhibit advantageous properties for this purpose. For a potential application in humans, the degradability of candidate biomaterials should be controllable, and correspond to the properties of the target tissue. In the case of the LGN discussed in the previous subsection (see Section 2.2.1), the biodegradability is controllable using the number of laminated gelatin-PCL layers. This is, however, just one possible means of influencing the degradation kinetics of a biomaterial. In this subsection, an innovative hydrogel is presented [176]. This hydrogel is cell-compatible, and its biodegradability is controllable by an external stimulus. Against this background, this hydrogel is also referred to as “smart hydrogel”. Chemically, it is based on multi-arm polyethylene glycol (PEG), to which gyrase B (GyrB) molecules are covalently bound. These GyrB proteins are additionally biofunctionalized with the above-described RGD integrin-binding motif. Upon addition of the aminocoumarin antibiotic coumermycin, dimerization of GyrB molecules takes place and induces hydrogel formation. The controlled dissolution of the hydrogel is made possible by the addition of the antibiotic novobiocin, which competes with coumermycin to bind to the GyrB molecules but does not support the dimerization. The mechanistic concept of the hydrogel, including RGD-biofunctionalization, coumermycin-induced formation, novobiocin-induced dissolution, and ZZ-functionalized, GyrB-mediated growth factor (FGF-7/KGF) incorporation (see below), is shown in Figure 3. 

In vitro tests on cell compatibility revealed that none of the hydrogel components had cytotoxic effects on monolayer-cultured oral cell types, namely primary GFs or HPV-16GM (=GKs). This applied to both the concentrations required for hydrogel formation and novobiocin-induced dissolution within four (20 µM novobiocin) or six hours (50 µM novobiocin). Upon the administration of 100 µM novobiocin, monolayer cells exhibited morphological alterations, which, however, was not the case for epithelial or connective tissue cells under in vivo conditions in a rat model. An immunohistochemical evaluation of tissue sections did also not reveal any signs of inflammation, as indicated by the lack of IL-6 expression [177]. This suggests that cells in monolayer cultures may be less resilient to novobiocin when compared to the complex tissue situation in vivo.

Generally, this stimulus-induced hydrogel dissolution is an important prerequisite for the release of bioactive substances from such biomaterials. To apply this principle, fibroblast growth factor 7 (FGF-7, formerly called keratinocyte growth factor (KGF)) was incorporated into the hydrogel, which is a strong inductor of keratinocyte proliferation [178]. To achieve the incorporation and controlled release, FGF-7 was fused to a fragment of the crystallizable region (*Fc region*) of an antibody, and GyrB was fused with the zinc finger domain (ZZ-domain) of protein A. The ZZ-domain functions as a receptor for the Fc region. Upon the addition of novobiocin, multi-arm PEG with GyrB-bound FGF-7 is released from the hydrogel network and the growth factor is subsequently able to bind nearby FGF receptors on keratinocytes. The bioactivity of the released FGF-7 was demonstrated in a subsequent keratinocyte proliferation assay. Regarding the potential application of this hydrogel in the context of oral and periodontal diseases, both the GFs and the GKs were shown to successfully populate the hydrogel until reaching confluence [176]. Cellular adhesion was, however, strictly dependent on the presence of the RGD sequence, since the hydrogels devoid of this binding motif failed to allow for cell adhesion and the spreading of both cell types [176,177]. GKs and GFs seeded on the hydrogels showed β1 integrin expression. α5β1 integrin recognizes the RGD sequence when it in a kinked conformation, as indicated by a study, which evaluated the activity and selectivity profile of ligands for RGD-binding integrins [179]. The results support the conclusion that the “smart hydrogel” is (i) cell- (in vitro) and biocompatible (in vivo), (ii) supports the adhesion and growth of different oral cell types in the presence of an RGD-binding motif, and (iii) can controllably release bioactive factors such as FGF-7 for the successful promotion of keratinocyte proliferation. 

Alternative hydrogel strategies for periodontal regeneration are, for instance, based on chitosan, which is the alkaline-treated form of the natural polymer chitin [180,181]. This is composed of stochastically distributed β-(1→4)-linked amino sugars D-glucosamine and its acetylated form, N-acetyl-D-glucosamine. By employing enzymatically solidified chitosan hydrogels, which were loaded with fluorescently labelled PDL cells, Yan and coworkers could show that these hydrogels did not have adverse effects in the surrounding tissue of rat intrabony periodontal defects. Of note, the hydrogels were largely degraded 4 weeks post-implantation. Moreover, treatment success was substantiated by a significant increase in functional PDL length compared with cell-free hydrogels. However, the contribution of the hydrogel-incorporated PDL cells to the treatment success could not be determined in detail, since no fluorescent dye signal was detectable after the 4-week observation period [182]. A more recent application based on chitosan-containing hydrogels consists of dental pulp SC-derived exosomes (exosomes are generally cell-derived extracellular vesicles; in this case, they are derived from dental SCs (DPSC-Exo)) that are incorporated into the hydrogels (DPSC-Exo/Chitosan) [183]. These hydrogels were able to speed up alveolar bone and periodontal epithelium healing in mice suffering from periodontitis. Mechanistically, healing was found to be associated with micro-RNA (miR)-1246 in DPSC-Exo, which exerts immunomodulatory effects by converting macrophages from a pro-inflammatory to an anti-inflammatory phenotype [184,185]. 

Periodontal tissue protective approaches, which employ protease-degradable (e.g., collagenase) hydrogels to release pathogenic bacteria-defeating substances, are reported in a paper published by Lin et al. Herein, the so-called hybrid hydrogels were created for synergistic periodontal antibacterial treatment with sustained drug release and a near-infrared (NIR, 808 nm)-responsive photothermal effect and evaluated for their efficacy against *PG* [186]. Following irradiation, the hybrid hydrogels comprising silica-coated mesoporous gold nanobipyramids (Au NBPs@SiO_2_), in conjunction with gelatin methacrylate and the antibiotic minocycline, exhibited antibacterial effects in an NIR light irradiation-dependent manner (Maximum: 1.2 W/cm^2^ NIR, antibacterial effect: 66.7%). In addition to antibiotic release, the photothermal effects of the Au NBPs@SiO_2_ hydrogel-innate particles improved with increasing NIR, progressively contributing to *PG* defeat, without seriously hampering cell growth in vitro [186]. This biomaterial-based antimicrobial treatment strategy is an interesting hybrid approach, which addresses oral pathogens as causatives of oral diseases with the help of a hydrogel. Another possibility of attacking such germs, which relies on different chemical principles, is discussed next. 

#### 2.2.3. Synthetic Mimics of Antimicrobial Peptides (SMAMPS) as a Strategy to Combat Oral Pathogens 

One major pathogenetic factor, which is common to many oral and periodontal diseases, is a dysregulated oral microbiome. Different bacterial species, e.g., *PG*, *TD*, *TF*, or *AA*, are known to actively cause, sustain, or at least passively permit inflammatory stimuli within the oral cavity [187]. For this reason, it is important to develop antimicrobial strategies that are, on the one hand, tissue-compatible and effective in combatting germs, and, on the other hand, do not cause antimicrobial resistances. The use of systemically administered antimicrobial chemotherapy is a controverse topic in periodontology [188]. However, innovative modes of drug delivery have been developed in recent years. One example is porous matrices made of gelatin or cellulose derivates, which are loaded with antibiotics (e.g., metronidazole). The local application of such matrices into deep periodontal pockets led to remarkable clinical effects, as represented by a reduction in the pocket depth and bleeding on probing [188,189].

When compared to classical antimicrobial chemotherapeutics, naturally occurring antimicrobial peptides (AMPs), show different mechanisms of action with high bacterial selectivity and a simultaneous reduction in drug resistances. One example of such AMPs are defensins, which are also found in saliva. Due to their advantages, much of the research effort has been concentrated on the synthetic reproduction of such AMPs, leading to polymeric therapeutics called synthetic mimics of antimicrobial peptides (SMAMPS). The antimicrobial effects of AMPs such as human β-defensin 3 (hBD3) [190] and SMAMPS are based on their amphiphilicity. These molecules have both hydrophilic and hydrophobic properties. Hydrophilic cationic groups are located opposite non-charged groups in the defensin-peptide/SMAMP-polymer. These positive charges interact with the negatively charged cell membranes of bacteria and do not interfere with the neutrally charged cholesterol containing outer-membrane leaflets of eukaryotic cells. Subsequently, the bacterial cells are killed, e.g., by toxic, pore-forming AMPs or via intracellular dysregulation of the cellular physiology [191]. 

In the context of AMP mimetics, the first promising results regarding oral pathogens, e.g., *Streptococcus mutans* (*S. mutans, SM*), that are etiologically involved in caries, were published by Beckloff et al. in 2007. In this study, the peptide mimetic *meta*-phenylene ethynylene (mPE), modeled after the amphiphilic magainin, an antimicrobial peptide from the skin of the African frog *Xenopus laevis,* exhibited antimicrobial activity (reduction in viable bacteria within an *SM* biofilm by 3 logs at [100 nM] mPE) at nanomolar concentrations. Based on structure–function analyses, the authors suggest that mPE, like its natural counterpart magainin, interferes with bacterial lipopolysaccharide (LPS) and DNA, acting as both a membrane-interfering molecule and an intracellular antibiotic [192]. mPE could also be shown to exert potent activity against biofilms of *AA* and *PG*, involved in chronic (*PG*) and aggressive (*AA*) periodontitis [193]. Moreover, in the same study, mPE [2 µg/mL] was demonstrated to be anti-inflammatory by reducing the activation of the nuclear factor ‘kappa-light-chain-enhancer’ of activated B-cells (NF-*k*B), as well as interleukin-1β (IL-1β)-induced IL-8 secretion [193].

**Figure 4:**
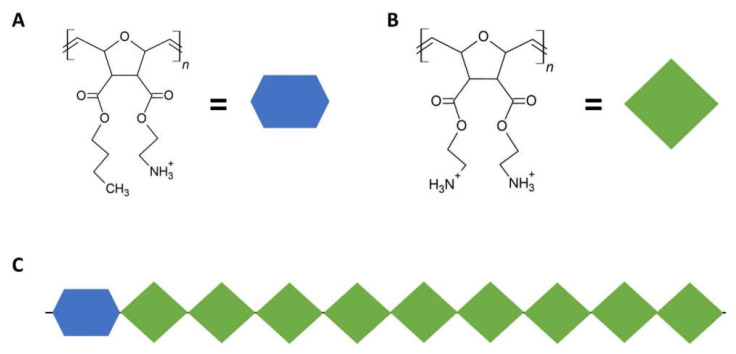
Principles of the design of synthetic mimics of antimicrobial peptides (SMAMPS). The SMAMPS described in the main text are based on a poly(oxanorbornene) backbone. (**A**) shows the chemical structure of unit 1 (U1; see main text), which is a monomer from a poly(oxanorbornene) structure. The side chains are composed of a butyl residue (left side) and a positively charged ethylamine (right side). (**B**) Unit 2 (U2; see main text) differs from U1 in that the butyl chain was replaced by another ethylamine residue, yielding two positive charges per monomer. (**C**) Schematic representation of a complete SMAMP composed of 1 U1 and 9 U2 subunits, which, therefore, has a considerable number of positive charges and leads to the selective killing of various bacteria. Details are given in the main text.

SMAMPS can also be designed from scratch. In one exemplary study, a modular approach was used to design the SMAMPS and to allow for sufficient amphiphilicity. A poly(oxanorbornene) backbone served as a chemical scaffold for further modification. Unit 1 (U1) was generated by attaching both a hydrophobic butyl group and a hydrophilic, positively charged ethylamine to the oxanorbornene group. A second unit (U2) with more hydrophilic properties was synthesized by replacing the alkyl residue with another ethylamine, yielding two positive charges. Due to this U1/U2-modular system, the generated SMAMPS could vary in their antimicrobial activity and cell compatibility. Generally, these SMAMPS-polymers had a molecular weight of approximately 3 kDa [194], which is in the range of natural AMPs, for instance, the periodontitis-relevant hBD3 (5.2 kDa) [195]. The modular system of SMAMPS is schematically illustrated in Figure 4. 

By choosing a ratio of 1 U1:9 U2, it could be shown that these rather hydrophilic SMAMPS exhibited a double selectivity in solution. First, they were selective for bacteria over erythrocytes and GFs, with the latter used as representatives of oral tissues in SMAMPS-compatibility studies. Second, they were more selective for Gram-positive than Gram-negative bacteria. Regarding the mechanism of SMAMPS-action, transmission electron microscopy (TEM) revealed that the bacterial envelopes were seriously damaged, independent of the bacteria’s Gram-status. Using TEM, this was demonstrated for Gram-positive *Staphylococcus aureus* (*SA*) and Gram-negative *Enterococcus faecalis* (*EF*) [194]. Both bacteria species are present in oral tissues, while *EF* is also associated with periodontitis [196,197]. This shows that the mode of action of the SMAMPS closely resembles that of natural AMPs. With respect to the above-mentioned antimicrobial resistances, Kuroda and Caputo could exemplarily show that the propensity of poly(methacrylate)-based SMAMPS in triggering bacterial (*Escherichia coli* (*ER*)) resistance is low when compared to “classical” antibiotics, such as the gyrase inhibitors Ciprofloxacin or Norfloxacin [198,199].

With respect to biomedical applications, e.g., the use of SMAMPS as coatings on biomaterials such as catheters or dental implants, it is mandatory to immobilize them on a surface. Therefore, a surface-attached (solid state) SMAMPS model was implemented. A benzophenone anchor group, which was cross-linked by pentaerithritol-tetrakis(3-mercaptopropionate) (=tetrathiol), was employed to immobilize the SMAMPS on model surfaces. Herein, comparable methods, as in the above-discussed study, were used to evaluate the cell compatibility. The impedance measurement-based cell index (CI) showed that GFs and GKs tolerated the chemically modified SMAMPS. By determining the minimum inhibitory concentration (MIC) for antimicrobial activity, SMAMPS’ efficiency was monitored. Therefore, it could be shown that, amongst others, surface-immobilized SMAMPS of the aforementioned 1 U1:9 U2 ratio [194] matched the in-solution activity, as described above. Moreover, to directly test for a biomedical application, medical-grade PDMS tubing was coated with the SMAMPS. In this applied approach, two coatings of 100-nm thickness were able to achieve an antibacterial efficiency (including *SA* and *EF*) of almost 100 % [61].

Another SMAPS approach addresses the above-mentioned periodontitis-relevant hBD3. Regarding this defensin, Scudiero and coworkers designed a cyclic β-defensin analog comprising 17 amino acids with one disulfide bond. This synthetic antimicrobial cyclic peptide (AMC) represents a combination of hBD1 and hBD3 by merging the internal hydrophobic domain of hBD1 and the C-terminal-charged region of hBD3. This was non-cytotoxic for immortalized human colorectal adenocarcinoma cells (Caco-2) and retained the antibacterial capacity of both native defensins, as shown by defeating bacteria including Pseudomonas aeruginosa (P. aeruginosa, PA), and the aforementioned ER and EF. In addition, AMC was also active against herpes simplex virus type-1 (HSV1) in a dose-dependent manner [200]. 

A more recent strategy to prevent pathogen-induced periodontitis arises from small-molecule inhibitors, which act as peptidomimetic compounds. They can inhibit PG interaction with oral streptococci and, thus, initial colonization of the oral cavity. Inhibition of this interaction was achieved by creating 2-(azidomethyl)- and 2-(azidophenyl)-4,5-diaryloxazoles with a full range of hydrophobic groups, which yielded so-called click products by reacting with substituted arylacetylenes [201,202]. The compounds mimic the antigen I/II streptococcus polypeptide, which serves as a docking region for the minor fimbrial antigen of PG, mechanistically inhibiting bacterial interaction. In a consecutive study, one compound could even be identified that inhibited PG-related virulence in an in vitro biofilm model and in a periodontitis mouse model [202]. 

In summary, this chapter on biomaterial-based oral research illustrates the broad spectrum of approaches to address diseases of this organ. The findings on biomechanics suggest that epithelial and STR, as well as tissue homeostasis, are dependent on certain optimal values of spacing patterns for cell adhesion and elastic moduli for epithelial morphogenesis. This means that biomaterials can carry the necessary biomechanical information for tissue formation themselves. Thus, the LGN-innate biomechanical information can be considered instructive for epithelial morphogenesis, but also for STR, as indicated by its successful population with GKs and connective tissue GFs. The idea of cell-instructive biomaterials was supported by studies on FAK shutdown in an EE model. These studies reveal that molecules such as FAK, which are involved in mechanotransduction, are addressed by the biomaterial’s biophysical information. Further, they demonstrate the overall importance of FAK for the regeneration of the gingival epithelium. Different materials, as exemplified by various gelatin-based nonwovens, could be shown to support the adhesion, survival, and proliferation of different periodontal cell types, as well as endothelial cells. The PEG-based, RGD-biofunctionalized smart hydrogels appear to be promising candidates for clinical use, since they are tunable regarding their assembly and disassembly. Furthermore, the possibility of incorporating pharmacologically active compounds render them an excellent tool for the controlled spatiotemporal release of drugs, which efficiently support tissue regeneration in oral diseases. Regarding regeneration, further options consist of using biodegradable chitosan-based hydrogels as carriers for periodontal cells or (pulp) SC-derived exosomes. Hydrogels comprising gelatin methacrylate, in conjunction with Au NBPs@SiO_2_ and an antibiotic, exert an NIR-irradiation-tunable synergistic antibiotic-photothermal impact on oral pathogenic bacteria, preventing the onset and persistence of inflammatory oral diseases such as gingivitis or periodontitis. Furthermore, these SMAMPS, which can be attached to biomaterials, offer a strategy to defeat oral pathogens, while lacking the disadvantages of classical antibiotics. The same holds true for the amphiphilic peptide mimetic mPE, which, compared to SMAMPS, bears the additional property of being anti-inflammatory. While SMAMPS and mPE interfere with the bacterial envelope, combinatory mimics of hBDs (hBD1/3) can inhibit periodontitis-contributing bacterial interactions at very early stages. 

Thus, the in vitro and in vivo reconstitution of a complex organ such as the periodontium may be enabled by the sophisticated combination of different materials to fulfill the biomechanical and biochemical needs of each cell entity or to protect oral tissues from pathogenic microbiota.

### 2.3. Cell-Based and Optogenetic Approaches: Perspectives in the Context of Oral Diseases

In the previous chapters, molecular alterations, and the biomaterial-based approaches derived in the context of oral diseases were discussed. This, however, does not represent the full spectrum of current and newly developed strategies to mitigate oral diseases. Against this background, this chapter deals with cell-based (Section 2.3.1) and optogenetic (Section 2.3.2) approaches, which integrate both cell biological and biomaterial-related principles. These selected possibilities offer new perspectives in regenerative oral medicine. 

#### 2.3.1. Cell-Based Approaches

To successfully treat inflammatory oral diseases such as gingivitis and periodontitis, a profound understanding of the molecular basis of these diseases is required. In vitro monoculture cell models and interactive co-cultures can be very valuable tools, as discussed above. The inflammation-associated molecules and molecular mechanisms that are uncovered can then be translated into diagnostic and therapeutic applications. For diagnostic purposes, the immortalized gingival keratinocyte cell line HPV-16GM (=GKs) (see Section 2.3) was used to detect the molecular processes that occur in the oral cavity as part of an inflammatory response. To simulate inflammation, GKs were exposed to the pro-inflammatory cytokine IL-1β for 24 h and the changes in gene expression were detected by epithelial-specific cDNA microarray analysis. Differentially expressed genes were assigned to different functional domains of cellular biology: (i) cell stress (several heat shock proteins (HSPs)), (ii) DNA repair (topoisomerase II), (iii) cell cycle and proliferation (cyclin B, cell division cycle 2 (cdc2; a cyclin-dependent kinase)), (iv) anti-pathogen response (IL-8, IP-10 (CXCL10, chemokine ligand 10), and MIP3a (CCL20, chemokine ligand 20)), (v) extracellular matrix and turnover (tenascin, laminins a5 (LAMA5), b1 (LAMB1), a3 (LAMA3), and c2 (LAMC2), as well as the matrix metalloproteinases MMP2 and MMP10), and (vi) angiogenesis (vascular endothelial growth factor (VEGF) and the related VEGF-C). Interestingly, the differential gene expression induced by IL-1β stimulation correlated with the translocation of NF-*k*B to the nucleus [203]. These findings show that similar molecular analyses can be used to identify the new candidate genes that are involved in inflammation-related oral and periodontal diseases. Further characterization of these candidates is the basis for their potential establishment as diagnostic oral disease markers. Regarding the myriad of transcription factors, NF-*k*B activity is intimately linked to periodontitis. In this context, the finding of inflammation-associated NF-*k*B nuclear translocation was confirmed in a recent work, in which the authors exposed PDLFs to bacterial LPS as an inflammatory stimulus [204]. 

As outlined in the introduction (see Section 1), SCs from the oral cavity, which largely exhibit the features of hMSCs, are a cornerstone of cell-based regenerative approaches for oral diseases including caries, periodontitis, and oral cancers. This broad field was recently reviewed by Yang et al. [205]. The expression of hMSC-featuring biomarkers was exemplified in a study on human dental follicle cells (DFCs), while distinct DFC-fractions, apart from embryonic and neural SC markers, expressed MSC-specific biomarkers (Notch homolog 1 (Notch1), mesenchyme-1 (STRO-1), cell surface receptor CD44, and HLA-ABC [(MHC) class I] as well as the IgG super-family member CD90) [206]. A more detailed analysis of the tissue-regeneration abilities of SCs derived from different oral tissues revealed that, for example, gingival mesenchymal SCs were superior to dental pulp-derived mesenchymal SCs with respect to cell functions such as (i) proliferation, (ii) migration, and (iii) induction of angiogenesis. While proliferation and migration were only analyzed under in vitro conditions, looking at colony-forming ability and wound scratch assays, angiogenesis was also analyzed in vivo by vessel formation in nude mice, following the grafting of SC-harboring Matrigel^TM^ constructs [207].

To exert their full regenerative potential, molecular interactions between SCs and their neighboring cells are indispensable. The interplay between dental MSCs and host tissue cells not only includes genuine periodontal cells, but also immune cells. This points to an SC-inherent immunomodulatory competence. This competence is exemplified by an in vitro study on PDLSCs, which were shown to modulate the immunological responses of neutrophils in a paracrine fashion. To this end, neutrophil-differentiated human promyelocytic leukemia HL-60 cells (HL60D) were treated with PDLSCs supernatants after PDLSC exposure to *PG* protein extracts. The response was determined by a significant increase in the HL60D recruitment rate and intracellular reactive oxygen species (ROS) compared to control conditions [208,209]. Moreover, osteogenic DPSCs were shown to inhibit the proliferation of peripheral blood mononuclear cells (PBMCs) when cocultured in vitro. Vice versa, levels of anti-inflammatory cytokines, e.g., transforming growth factor-β (TGF-β), increased [208,210]. 

Against this background, studies were conducted on the biophysics and interactions of SCs with other periodontal cell types. With respect to periodontal SCs, studies on the effects of the environmental biomechanical stiffness on cell behavior are very common, as exemplified by a current publication by Liu et al. In this, the authors showed that increasing substrate stiffness promotes the proliferation and osteogenic phenotype of PDLSCs. This was indicated by the increased expression of alkaline phosphatase (ALP), osteopontin (OPN), osteocalcin (OCN), bone morphogenic protein-2 (BMP-2), and Runt-related transcription factor 2 (*RUNX2*, also known as core-binding factor subunit alpha-1 (CBF-alpha-1)) [211]. Contrary to this, studies on the spacing of anchor points for SCs’ cell adhesion are rare. However, it could be shown that the micropatterns, and thus the provision of potential adhesion points for FN-biofunctionalized PDMS microcolumns, determine the adhesion and morphogenesis, as well as the proliferation and differentiation of hMSCs. Smaller column spacings of 5 µm and 7 µm favored cell attachment and cell spreading/morphogenesis, when compared to spacings of 9 µm and 11 µm. At the cell behavioral level, the hMSCs’ proliferation continuously decreased with increasing distances. Regarding differentiation, typical mesenchymal SC-associated genes were expressed at higher levels in cells grown on micropatterns with large pillar distances, preferentially 9 µm and 11 µm. This was revealed by an analysis of leukemia inhibitory factor (LIF), basic fibroblast growth factor (bFGF), nucleoside diphosphate-linked moiety X motif 6 (NUDT6), and nestin, suggesting the preservation of the SC character under these environmental conditions [212]. These findings show that basic SC functions, such as adhesion, morphogenesis, proliferation, and the SC character per se, also depend on biophysical cues, as summarized at the end of Section 2.2. 

Cell behavior in its natural environment, however, is even more complex. When in vitro-cultured SCs are transferred to the respective periodontal tissue for the purpose of tissue regeneration, the transplanted SCs come into direct or indirect contact with the surrounding cells of the host tissue and interact with them. This interaction implies that SCs influence the growth and differentiation properties of host cells and vice versa. Therefore, it is important for the SC-based regeneration of periodontal tissues to broaden the knowledge of the phenotypic or cell behavioral effects of such interactions and the molecules involved. To address this important aspect of intercellular crosstalk, interactive cocultures of hMSCs with human (h) hGFs, hPDLFs, and osteoblasts of alveolar bone (hOA), were established. The resulting hMSCs behavior was examined. The analyses revealed that these oral cell types affected hMSCs’ behavior, in that the SCs showed a reduction in both proliferation and typical SC marker gene expression (e.g., POU domain class 5 transcription factor 1 (POU5F1), transcription factor homeobox protein Nanog, and SC growth factor receptor, also known as tyrosine protein kinase kit (c-kit)) within a 2-week observation period. This proliferation-reducing and differentiation-inducing effect was most pronounced when hMSCs were cocultured in the presence of hOA and was less pronounced in the presence of hGFs and hPDLFs. hMSCs reduced the number of apoptotic cells, regardless of the cocultured oral cell type [213]. These findings show that hMSCs and the respective periodontal host cell entities mutually influence each other regarding cell behavioral features including proliferation, hMSC stemness, and cell survival. Thus, the results obtained from this kind of interactive coculture are form the basis of predictions regarding stem and host cell behavior in hMSC-based oral tissue regeneration. 

In further studies with these cocultures, it could be demonstrated that hMSCs elicited the expression of markers of osteogenic differentiation, e.g., OPN, OCN, RUNX2, and collagen 1a1 (COL-1a1), in the three cell types described above. Additionally, 3D-hMSC-periodontal cell cocultures resulted in increased bone matrix production and a higher presence of mineralization nodes compared to control cultures without hMSCs. The expression of the osteogenic phenotype in the co-culture decreased from hOAs to hGFs and hPDLFs [214]. These findings indicate that hMSCs are potentially capable of stimulating the osteogenic phenotype to varying degrees in oral host cells. They also show that different periodontal cells have a different degree of inherent plasticity concerning osteogenic differentiation. These insights provide valuable clues regarding the use of hMSCs for oral bone regeneration. 

In this context, the cell behavioral effects of hMSCs on hOAs were further analyzed. In hMSC-hOA-cocultures, hMSC-derived VEGF release induced the chemoattraction of hOAs. This mechanistic connection was confirmed by chemoattraction assays and employment of the VEGF receptor inhibitor 3-[4-(dimethylamino)benzylidenyl]indolin-2 (=SU4312), with the latter completely abolishing hOA chemoattraction towards hMSCs [215]. This finding shows that hMSCs may contribute to periodontal/oral bone regeneration, not only through the induction of the osteogenic phenotype in oral host cells, but also by attracting hard tissue-forming cells to sites of bone regeneration. Moreover, this study supports the notion that oral tissue/periodontal tissue-derived SC may exert a regenerative action on damaged oral/periodontal tissues via secreted bioactive molecules, as suggested by the observed chemo-attractive capacity of hMSC-derived VEGF. In the context of oral bone homeostasis, this suggestion is backed up by an in vitro study on murine MSCs (mMSCs), which were cocultured together with osteoclast precursor cells (RAW264). Here, the authors found that the chemokine C-C motif chemokine ligand 2 (CCL2) secreted by the mMSCs was responsible for the chemotaxis of RAW264 cells in a Boyden chamber assay [216]. 

The importance of soluble factors such as VEGF as determinants of cellular differentiation and behavior is a long-established concept. This notion is increasingly important for oral and periodontal tissues. It could be shown that the medium supernatants obtained from oral/periodontal SCs (also designated as a conditioned medium (CM)) can support periodontal tissue regeneration. The regenerative potential of CM was recently reviewed by Lin et al. [217], and exemplified in a study by Qiu and coworkers. Herein, collagen membranes harboring the CM of gingival mesenchymal stem cells (GMSCs) and PDLSCs were able to effectively support periodontal tissue regeneration in a rat periodontal defect model. Methodically, regeneration was detected by comparing newly formed periodontal ligament and alveolar bone in CM-treated versus non-treated animals [218]. In a further rat periodontal defect study, employing the CM of PDLSCs and GFs, only the PDLSC-CM was able to support periodontal tissue regeneration, as indicated by newly formed bone, whereas the GF-CM failed to do this. PDLSC-CM proteome analysis revealed a complex mixture of components, including extracellular matrix proteins, enzymes, angiogenic factors, growth factors, and cytokines. Thus, many potential candidates for this so-called secretome may be involved in the actual tissue regeneration process. In addition, the authors could show that PDLSC-CM treatment led to a decrease in periodontal inflammation, as indicated by decreased mRNA levels for tumor necrosis factor alpha (TNF-α) [219]. These findings underscore the SCs’ various mechanisms of action influencing and directing their neighboring cells in the context of tissue regeneration.

Regarding SC-based regeneration, another interesting question arises: is periodontal regeneration also possible without the selected SC populations? To answer this question, an alveolar bone regenerative approach that is completely devoid of preselected SCs was established. Herein, mixed cell populations were created from oral tissues, namely, the alveolar bone, the PDL, and the gingival connective tissue. The tests for biomarkers of progenitor and SCs c-kit, STRO-1, and melanoma cell adhesion molecule (MCAM/CD146), an accepted marker for stem/progenitor cells in combination with STRO-1 in periodontal cells [220], only showed a marginal proportion of progenitor and SCs, regardless of the cell fraction. After culturing the three cell populations in a medium specialized for osteogenic differentiation, classical osteogenic biomarkers (e.g., bone gamma-carboxyglutamate protein (BGLAP/Osteocalcin), RUNX2, Osterix (OSX = transcription factor SP7) and ALP) were increased in the cell population of alveolar bone and PDL at the gene expression level, while they were downregulated in the gingival connective tissue fraction (GCTF). While the increase in biomarker expression in the bone fraction was not surprising, the results in the PDL cell fraction (PDLCF) show that the osteogenic phenotype is inducible. Moreover, a comparison of PDLCF with GCTF revealed a much higher matrix mineralization and ALP activity in PDLCF, as detected by the quantification of alizarin red stain and enzyme activity [221]. These data suggest that PDL cells do not require SC input to contribute to bone regeneration and may, therefore, be promising candidates in the development of prospective cell-based therapy concepts.

Another strategy for prospective SC-free alveolar bone regeneration is the use of osteoblasts. These cells can be stimulated under in vitro conditions so that they are pushed as much as possible towards bone formation, i.e., they are preconditioned. Due to their growth and differentiation behavior, these preconditioned cells could be promising candidates for in vivo re-transference to efficiently support bone formation at the implantation site. To be as independent as possible from expensive growth factors and cytokines, as well as hormones or xenobiotics, during this preconditioning, a poly(methyl methacrylate) (PMMA)/polycarbonate (PC)-based microchip with an FN biofunctionalization was chosen as a platform. The latter is also suitable for 3D cell culture [222]. Osteoblasts inoculated into the FN-functionalized microcavities (300 µm × 300 µm × 300 µm; width × length × depth) of the chip revealed homogenous cell adhesion and morphogenesis, as well as a high vitality and growth, as indicated by scanning electron microscopy (SEM) and dye-based live/dead-staining for up to 14 days of culture. This was accompanied by significantly increased gene expression levels for osteonectin (ON), OCN, and ALP in comparison with matched conventional 2D-monolayer cultures. In addition, the formation of multilayered osteoblast aggregates of a uniform size within the microcavities was detected by Azur II staining from day 7, concomitant with the intercellular deposition of ECM bone matrix constituents, including OCN, ON and FN [223]. In current in vitro and in vivo bone-healing studies, FN was shown to contribute to fracture healing. Lee and coworkers reported an experimental setup in which the type III domains of FN (domains 9 and 10) were fused to elastin-like polypeptides (FN-ELPs). When using these FN-ELPs as a cell culture dish coating, the osteogenic differentiation of hMSCs is triggered. hMSCs exhibited elevated ALP and mineralization activity in conjunction with an increased gene expression of OPN and COL-1 [224]. Under in vivo conditions, a coating of beta-tricalcium phosphate (β-TCP) particles (size 0.25–1 mm) with an FN solution (1 g/L) significantly increased the guided formation of bone within 8 weeks in a rat calvaria critical-sized defect model [225]. This renders FN a suitable candidate for prospective bone fracture healing in the case of oral diseases.

In a further approach using the previously described PMMA/PC-microchip, biomechanical cues, such as interstitial biomechanical fluid flow through the lacunar–canalicular system (LCS), were imitated. LCS flow is an important environmental cue in hard tissue/bone homeostasis and remodeling [226]. The optimization of LCS flow has been analyzed in a brand-new article published by Wang and coworkers, which demonstrates, with the help of a multiscale model, that a few load cycles with rest insertion, high strain magnitude and rate support LCS flow within the osteocyte LCS [227]. Concerning the importance of LCS flow for bone tissue, osteoblasts of the alveolar bone were inoculated into the microcavities of the 3D-chip and cultivated under static and fluid flow conditions in perfusion bioreactors for 7 days. An SEM-based comparison of the two culture conditions revealed that the osteoblasts differed significantly in terms of morphogenesis. The cells cultivated under fluid flow conditions were exclusively reorganized into a rotund, osteon/bone-like tissue that consisted of densely packed, multicellular, three-dimensional cell aggregates. As indicated by time-lapse microscopy, the formation of these mulberry-like cell aggregates occurred within the first 24 h. Cell aggregate formation was also accompanied by higher ECM gene expression and the expression of bone differentiation-associated genes (COL-1, OCN, ON and ALP) after 7 days of culture. Mathematical modeling of the fluid flow conditions within the microchips revealed that this ranged from 8 to 32 µm/s, which is roughly in the range of the flow velocity of 24–84 µm/s for the osteocyte process membrane of native bone tissue [228]. These results suggest that, on the one hand, the culture can precondition osteoblasts in the direction of bone-like structures under 3D conditions, and, on the other hand, fluid flow in 3D cultures represents a further trigger to stimulate the bone micro-tissue formation of osteoblasts in vitro. 

#### 2.3.2. Optogenetic Strategies 

Cell interactions and biomaterials can induce a specific cell-behavioral response in target cells (see Section 2.2 and Section 2.3.1). This is, however, a complex process, whose specific steps and interactions are not always well understood. Many applications and research questions in regenerative medicine make the more specific control of cellular responses desirable. Optogenetics is a subtle concept to induce the spatiotemporally controlled expression of regeneration-promoting genes in target cells. To this end, light-inducible transgene expression systems are used. 

A red/far-red light-triggered on/off gene switch is presented to demonstrate the possible mode of operation of such a system. This was tested in diverse mammalian cell types in vitro for its general applicability. Subsequent in vivo testing aimed for spatially controlled angiogenesis in a chicken embryo model [229]. 

“*Switch-on*” *of the optogenetic system*: The first step is the transfection of target cells, e.g., primary human umbilical vein endothelial cells (HUVEC), for the controlled expression of a target gene, e.g., VEGF. Illumination with red light (660 nm) leads to the isomerization of the chromophore phycocyanobilin B (PCB, purified from the cyanobacterium *Spirulina*), which is bound to the intracellularly expressed phytochromobilin (PhyB). Induced by the isomerization-dependent conformal change in PhyB, the chromoprotein interacts with a split transcription factor comprising two subunits, U1 and U2. The PhyB-U1-U2 complex represents a functional transcription factor, which binds via a distinct element (DE) to a certain operator region (CR) of a vector DNA. Complex binding to CR recruits RNA polymerase II and induces the transcription of the gene of interest (GOI) within a so-called response vector (RV). In an exemplary study, genes such as human VEGF, which is supportive of angiogenesis during wound-healing and tissue regeneration, were used. For oral tissues, VEGF derived from saliva was recently reported to support wound-healing in patients in response to tooth extraction, which qualifies this optogenetic system for potential use in the oral cavity [229]. 

**Figure 5:**
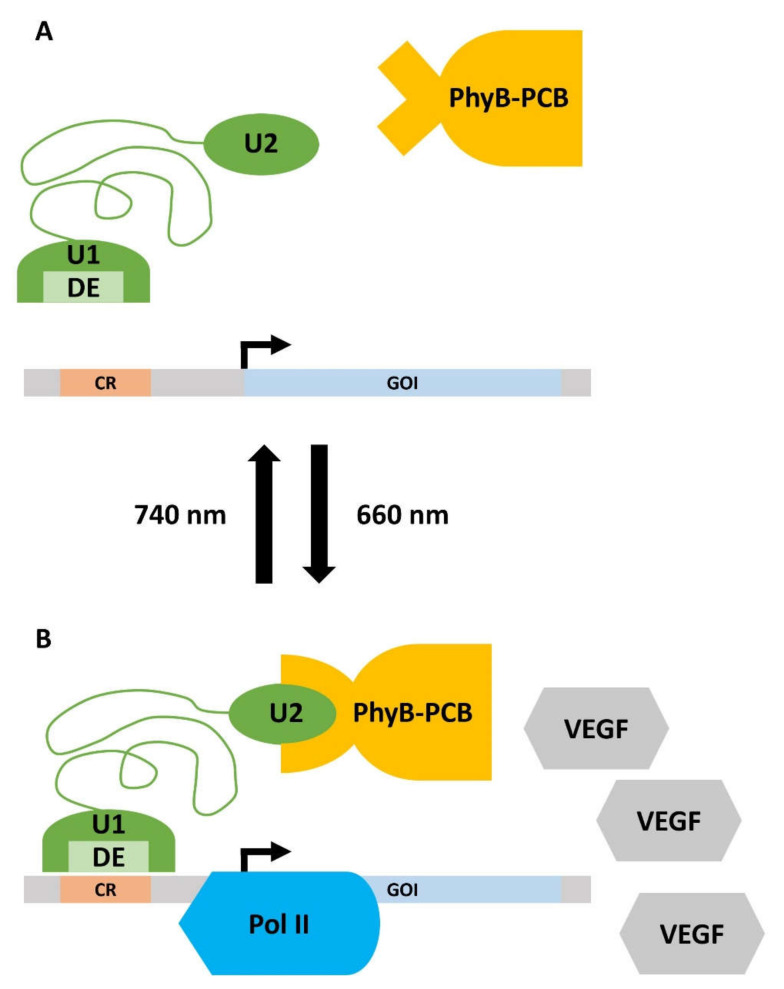
Working principle of the optogenetic gene expression switch. (**A**) Upon irradiation with far-red light (740 nm), the gene expression is turned off. The phycocyanobilin B (PCB), which is bound to phytochromobilin (PhyB), is in a closed conformation and cannot bind to unit 2 (U2) of the split transcription factor (green). Thus, the distinct element (DE) of unit 1 (U1) of the transcription factor does not interact with the certain operator region (CR) on the response vector. Consequently, there is no detectable expression of the gene of interest (GOI). (**B**) Illumination with red light (660 nm) leads to the isomerization of PCB, which induces a conformational change in the PhyB-PCB complex. PhyB-PCB can now recognize U2, which activates the split transcription factor. DE recognizes CR and recruits RNA Polymerase II (Pol II) to the promotor of the response vector. The GOI, in this case, vascular endothelial growth factor (VEGF), is now expressed. Details are given in the main text.

“*Switch-off*” *of the system*: Upon illumination with far-red light (740 nm), the PhyB-U1-U2 complex dissociates, thereby silencing the GOI expression within the RV [230]. The functioning of the optogenetic system is shown in Figure 5. 

This optogenetic system was first applied for tissue engineering purposes in a chicken chorio-allantoic-membrane (CAM) assay. Herein, the red light-controlled system with human VEGF as GOI was transfected into Chinese hamster ovary cells (CHO-K1, ATCC CCL 61). Subsequently, the cells were incorporated into a PEG-based hydrogel prior to placement onto CAM. In response to 660-nm red-light illumination, CAMs of chicken embryos exhibited significantly stronger human VEGF-mediated angiogenesis compared to controls, which were subjected to *switch-off* conditions [230]. 

Of interest, the spatiotemporally controlled gene expression using the optogenetic *switch-on* and *switch-off* mechanisms can also be used to simultaneously regulate multiple target genes within one cell. This is possible via the simultaneous use of different excitation wavelengths (multichromatic approach). In a proof-of-principle study, three optogenetic gene expression systems could be transferred to a single CHO-K1 cell, while the optical switches responded to the wavelengths of 311 nm, 465 nm, and 660 nm. In this case, the chosen GOIs were blue light-inducible firefly luciferase (FLuc, 465 nm), red light-inducible secreted alkaline phosphatase (SEAP, 660 nm; important for hard tissue formation also in oral tissues such as alveolar bone), and UVB-inducible proangiogenic angiopoietin 1 (Ang1, 311 nm). Notably, in the case of UVB, cells were pulsed for induction to avoid cytotoxicity [231]. The results show that the optogenetic GOI(s) system(s) described here can be transferred to different cell types, strongly suggesting their future implementation in periodontal cells. This approach could potentially promote the regeneration of respective tissues in response to oral diseases or trauma, since the oral cavity, in comparison with other organs, can easily be reached by light sources. 

In this context, Huang and coworkers recently published a promising study. Herein, the transcription factor Lhx8, which is also important in the embryonic development of craniofacial tissues such as bone and teeth [232], was introduced into bone-marrow-derived stem cells (BMSCs) in a UV-inducible optogenetic system. In vitro studies on human oral pulp stem cells (hDPSCs) have shown that Lhx8 promotes their proliferation, and Lhx8 overexpression in hDPSCs yielded the attenuation of osteogenesis [233]. Indeed, the optogenetic induction of Lhx8 expression in BMSCs first stimulated their early proliferation and, later, their differentiation. In a rat calvaria critical-sized bone defect model, the UV-pulse-driven induction of Lhx8 expression in BMSCs-harboring Poly(lactic-co-glycolic acid) (PLGA) scaffolds resulted in a significant increase in bone formation in vivo [66]. With these experiments, the general feasibility of an optogenetic-driven regenerative approach for hard tissues, as also found in the periodontium, was shown.

Taken together, apart from the environmental stiffness, the patterning of adhesion points additionally appears to be decisive for SC morphogenesis and behavior. The interactive cocultures of SCs and periodontal cells (i) are useful in determining stem and host cell behavior in hMSC-based oral tissue regeneration. (ii) They also show that different periodontal cells have a different degree of inherent plasticity concerning osteogenic differentiation. Moreover, (iii) they show that hMSCs may contribute to oral bone regeneration not only by the induction of the osteogenic phenotype in oral host cells, but also by attracting hard tissue-forming cells to bone regeneration sites through chemotaxis. 

As an alternative to the direct grafting of SCs, CM, i.e., the secretome of periodontal SCs, appears to be promising due to its ability to support alveolar bone formation. Regarding alveolar bone loss in response to oral diseases, regeneration possibilities not only arise from SCs, but also from periodontal ligament cells, since they can be pushed towards the osteoblastic phenotype. Alternatively, the promising results of PMAA/PC-microchip-cultured osteoblasts suggest that microtissue-preconditioned alveolar bone osteoblasts are candidates for prospective retransfer therapies in the clinic. 

Another aspect critical for tissue reconstitution is the spatiotemporal control of gene expression. Here, optogenetic *switch-on* and *switch-off* of transcription systems of one or multiple GOIs within a single target cell pave the way for light-controlled strategies, which enable targeted gene expression during future oral tissue regeneration. 

## 3. Conclusions

Despite the many advances in the field of molecular research regarding oral diseases and related biomaterials, there is still an enormous need to translate this knowledge into diagnostic and therapeutic applications. Many oral diseases are based on the molecular changes caused by pathogens or external noxae, for instance, the consumption of alcohol or tobacco. To obtain a better understanding of the molecular pathophysiology, suitable in vitro cell culture systems are required, which allow for the identification and characterization of candidate molecules for diagnosis and therapy. This knowledge is indispensable in the functional and structural restoration of damaged tissue, such as during periodontitis or oral carcinoma. Thus, biomaterials that carry the biophysical information for tissue regeneration and enable the spatio-temporally controlled release of tissue regenerative bioactive molecules, such as cytokines or growth factors, are required. Apart from targeting host cells, therapeutics directed against oral pathogens are the cornerstone of oral medicine. Mimics of biomolecules, such as oral defensins, are useful candidates to combat the bacterially induced destruction of periodontal tissues, as they largely prevent bacterial resistance to antibiotics. 

Concerning the cell-based strategies for oral tissue regeneration, SCs from periodontal tissues are important for translational research. In this context, SC behavior can be elucidated by in vitro coculture experiments, which reveal the mutual influence of SCs on oral tissue cells and vice versa. Moreover, SCs have both immunomodulatory and anti-inflammatory actions. Although their beneficial impact on humans seems to be promising, the fates and functions of SCs after transplantation still need to be elucidated in more detail. Furthermore, the exact mechanisms of tissue regeneration through SCs is partly unknown and, therefore, are an open research question [234]. Further SC-related regenerative options lie in SC-derived CM and exosomes. In addition, experimental approaches to cell preconditioning with in vivo-relevant 3D culture systems (so-called bioreactors) provide further options in the regeneration or augmentation of oral tissues. 

Optogenetics is a very young but promising research field for the spatio-temporally controlled expression of one or more target genes. This principle can be applied to support tissue regeneration in the oral cavity. Although this method has largely been applied experimentally to date, its benefits to regenerative medicine and dentistry are already foreseeable [67].

These key messages beautifully illustrate the broad spectrum of current oral research, ranging from the fundamental principles of cell culture studies to sophisticated biomaterial-based treatment approaches to oral regeneration.

## 4. Methods

This narrative review aims at summarizing and discussing selected research on the molecular principles of oral biology, as well as related biomaterial applications. The primary literature was retrieved from Medline and Web of Science databases using a semi-systematic research approach. First, research works dealing with oral squamous cell carcinoma cell lines from the last 5 years were collected and screened for their molecular characterization in terms of cytokeratin expression patterns. Illustrative examples were selected to compare the advantages and disadvantages of current approaches. Additionally, forward citation searching was applied to own research work in the field to identify related articles, which are additionally relevant to in-depth discussions on oral epithelial homeostasis and carcinogenesis. As a next step, the same search strategy was applied to biomaterial-based regenerative approaches. Due to the immense amount of research work in this particular area, the search terms were specified as three domains: (i) nonwoven-based, (ii) hydrogel-based, and (iii) antimicrobial approaches. Based on the current literature and own research work, a forward and backward citation search yielded relevant articles, a selection of which are presented in this review, based on their illustrative power. Additional articles with interesting new principles or advancements in basic principles, polymer modifications, optogenetics, etc., were included to provide a broad overview of the whole topic and illustrate the developments in the field of translational oral medicine.

Based on this strategy, this review is conceived in the form of a literature review that maps the state of research on a specific topic, which also includes own research, and critically examines this along with the current literature.

## Data Availability

Not applicable.
